# Objective Skin Property Assessment for Adaptive Fingerprint Image Acquisition and Processing

**DOI:** 10.3390/jimaging12100516

**Published:** 2026-10-15

**Authors:** Martin Drahanský, Petr Malaník, Jaromír Štěpánek, Tomáš Vokálek, Pavel Borčevský

**Affiliations:** 1 Department of Criminalistics, Faculty of Security Law, Police Academy of the Czech Republic in Prague, Lhotecká 559/7, 143 00 Prague, Czech Republic; stepanek@polac.cz; 2 TrendBit a.s., Strmá 2811/63, 616 00 Brno, Czech Republic; malanik@trendbit.cz; 3 Department of the Criminalist Technology and Expertise, Military Police, Rooseveltova 620/23, 160 05 Prague, Czech Republic; tomas.vokalek@mo.gov.cz; 4 Department of Criminal Law, Faculty of Security Law, Police Academy of the Czech Republic in Prague, Lhotecká 559/7, 143 00 Prague, Czech Republic; p.borcevsky@polac.cz

**Keywords:** fingerprint recognition, fingerprint image quality, skin properties, multisensor skin assessment, post-mortem fingerprints, adaptive acquisition, image enhancement, artificial intelligence

## Abstract

Fingerprint image quality depends not only on the acquisition technology but also on the physical condition of the examined skin. This study presents a portable multisensor system for objective skin assessment prior to fingerprint acquisition and evaluates its use under real forensic conditions. The system measures skin color, surface temperature, galvanic skin response, and relevant environmental parameters. Measurements were performed on 50 deceased individuals and complemented by fingerprint acquisition using multiple sensing technologies. The results revealed substantial variability in post-mortem skin properties, while no individual measured parameter provided a sufficiently strong relationship with fingerprint image quality to serve as a universal predictor. These findings motivate a skin-aware acquisition framework in which multisensor information may support future decision-making for skin conditioning, sensor selection, and controlled image processing. Illustrative generative-enhancement cases further demonstrate that improved fingerprint image-quality scores do not necessarily indicate preservation of identity-bearing ridge information.

## 1. Introduction

Fingerprint recognition is one of the most established biometric identification technologies and is widely used in forensic, law-enforcement, access-control, and civil identification applications. Despite the maturity of fingerprint recognition algorithms, their performance remains strongly dependent on the quality of the acquired fingerprint image. Poor ridge visibility, insufficient contrast, saturation, missing regions, and other acquisition artefacts can substantially affect subsequent feature extraction and matching [1,2]. Consequently, reliable fingerprint recognition depends not only on the performance of the recognition algorithm but also on the conditions under which the biometric sample is acquired.

Fingerprint image quality is influenced by the characteristics of the acquisition system and by the physical condition of the examined skin. In particular, *skin moisture*, *dryness*, *temperature*, and other *physiological characteristics* have been shown to correlate with *fingerprint image quality*, with the magnitude and nature of these effects depending on the sensing technology [3]. Optical, capacitive, multispectral, and contactless acquisition systems interact with the finger through different physical principles and consequently differ in their sensitivity to moisture, electrical conductivity, surface temperature, skin color and optical properties, contact pressure, and geometrical deformation of the friction-ridge surface. The same physical condition of a finger may therefore result in substantially different fingerprint image quality when different acquisition technologies are used.

Despite this dependence, conventional fingerprint acquisition systems generally provide little or no objective information about the physical condition of the skin before acquisition. Quality assessment is typically performed only after the fingerprint image has been captured, when an unfavorable interaction between the skin and the selected sensing technology has already affected the acquired sample. This limitation motivates a pre-acquisition approach in which relevant properties of the skin and the surrounding environment are objectively characterized before fingerprint capture. Such information may provide additional context for selecting an appropriate sensing technology, determining whether controlled skin conditioning is beneficial, deciding whether reacquisition is necessary, and adapting subsequent image processing.

To investigate this concept, this paper presents a portable *multisensor skin assessment device* consisting of a handheld unit and a dedicated multisensor measurement head. The system simultaneously measures skin color, surface temperature, galvanic skin response, and blood oxygenation together with ambient illumination, temperature, and relative humidity. The technical solution is protected by Czech utility model CZ 39522 U1 and was developed with biometric applications among its intended use cases [4]. The integration of complementary sensing modalities within a single portable platform enables quantitative characterization of several physical properties of the same local skin region and provides a common basis for relating the measured skin state to subsequent fingerprint acquisition.

The central concept of the present work is therefore to treat the skin not as a constant acquisition interface, but as a variable physical medium whose current condition may influence the interaction with a fingerprint sensor. Skin and environmental parameters measured before capture can be associated with fingerprints acquired using different sensing technologies and with the resulting fingerprint image quality. Objective quality measures, such as NFIQ 2, provide a standardized means of evaluating the utility of the acquired fingerprint samples [5]. In a future skin-aware acquisition framework, the measured contextual information could support decisions concerning skin conditioning, sensor selection, and reacquisition, and could additionally provide input for adaptive conventional or artificial-intelligence-based image processing.

The use of adaptive or generative image processing introduces an additional requirement that is particularly important in biometric and forensic applications. Improvement in visual appearance or in an objective image-quality score is not sufficient if the processing alters identity-bearing fingerprint information. Any enhancement intended for biometric use must preserve the original ridge topology and minutiae and must not introduce, remove, or displace biometric features unsupported by the acquired fingerprint [6]. Accordingly, the present study distinguishes between improvement of fingerprint image quality and preservation of biometric fidelity and uses this distinction when discussing the potential role and limitations of AI-based fingerprint enhancement.

The main contributions of this work are:

- First public presentation and experimental evaluation of the developed portable multisensor unit for objective pre-acquisition assessment of skin and environmental conditions;
- Experimental investigation of the relationship between objectively measured skin properties, fingerprint image quality, and different fingerprint sensing technologies under post-mortem forensic conditions;
- Introduction of a skin-aware acquisition concept in which objective pre-acquisition measurements may support skin conditioning, sensor selection, reacquisition, and subsequent adaptive image processing;
- Analysis of the limitations of individual skin parameters as predictors of fingerprint image quality and identification of the need to incorporate morphological characteristics of the friction-ridge surface into future decision-support approaches;
- Discussion of the potential and forensic limitations of AI-based fingerprint enhancement, with particular emphasis on the distinction between improved image-quality metrics and preservation of identity-bearing biometric information.

The remainder of this paper describes the relevant fingerprint sensing and skin-characterization principles, the design and implementation of the developed multisensor unit, its experimental evaluation under post-mortem forensic conditions, and the implications of the obtained results for future skin-aware fingerprint acquisition and processing.

## 2. Background and Related Work

Fingerprint image quality results from the interaction between the sensing technology, skin properties, acquisition conditions, and subsequent processing. Since various sensing principles respond differently to the physiological and optical conditions of the skin, these factors should be considered jointly. This section therefore briefly reviews fingerprint sensing technologies, relevant skin properties, image quality assessment, and adaptive acquisition and processing approaches as the background for the proposed skin-aware framework.

### 2.1. Fingerprint Acquisition Technologies

Fingerprint acquisition technologies exploit different physical interactions between the finger and the sensing system, and consequently differ in their sensitivity to skin condition and acquisition environment [7]. In this work, particular attention is given to optical, capacitive, and contactless sensing, as these technologies are widely used and are directly relevant to the experimental evaluation.

*Optical sensors* typically form a fingerprint image from differences in light reflection between ridges and valleys. A well-established principle is frustrated total internal reflection (FTIR), where ridge contact modifies the optical conditions at the platen surface. Optical acquisition can provide large sensing areas and high-quality images, but may be influenced by surface contamination, insufficient contact, excessive moisture or dryness, and the optical properties of the skin [7,8]. Of particular interest are optical systems employing near-infrared (NIR) illumination, which can provide additional information from subsurface skin structures and reduce dependence on purely visible surface characteristics. Multispectral systems extend this principle by combining multiple wavelengths, illumination geometries, and/or polarization states to improve acquisition under challenging skin conditions [9].

*Capacitive sensors* reconstruct the ridge–valley pattern from local differences in capacitance between the finger and an array of sensing elements. Their compact size makes them suitable for portable and embedded biometric systems. Since the measurement depends on electrical coupling and ridge-to-sensor distance, image quality can be affected by skin conductivity, moisture, dryness, contact pressure, and contamination [7,8]. This dependence is particularly relevant to the present study because electrical skin properties are among the parameters measured by the proposed multisensor unit.

*Contactless fingerprint acquisition* avoids direct mechanical interaction with a sensing surface and captures the finger using one or more imaging devices. Systems range from 2D photographic acquisition to multi-view and 3D approaches based on stereo vision, structured light, or photometric methods [10,11]. Contactless acquisition eliminates platen-related contamination and pressure-induced deformation, but introduces other challenges, including illumination variability, perspective and scale changes, depth of field, and reconstruction of the curved finger surface.

A number of additional sensing principles have also been investigated, including *thermal*, *ultrasonic*, *pressure/piezoelectric*, *electric-field/electrostatic*, MEMS-based, and specialized optical or fiber-optic approaches [7,12]. These technologies are not discussed in detail here, as the present study focuses primarily on sensing principles represented in the experimental evaluation. The diversity of available technologies nevertheless supports the central premise of this work: the same skin condition may interact differently with different fingerprint acquisition principles, motivating objective skin assessment before sensor selection and image acquisition.

### 2.2. Skin Properties Relevant to Fingerprint Acquisition

The fingertip skin forms the physical and optical interface between the subject and the fingerprint acquisition system. Although the friction ridge pattern is defined by the epidermal ridge–valley structure, its representation in the acquired image also depends on the current condition of the skin. Relevant factors include *surface moisture, pigmentation and optical properties, temperature, elasticity, contamination, and the condition of the outer epidermal layers*. These parameters may vary with environmental conditions, perspiration, washing, occupation, physiological state, and post-mortem changes. Previous experiments have investigated moisture, oiliness, elasticity, and temperature across different fingerprint sensing technologies, demonstrating substantial variability associated with both skin condition and sensing principle [3].

**Figure 1:**
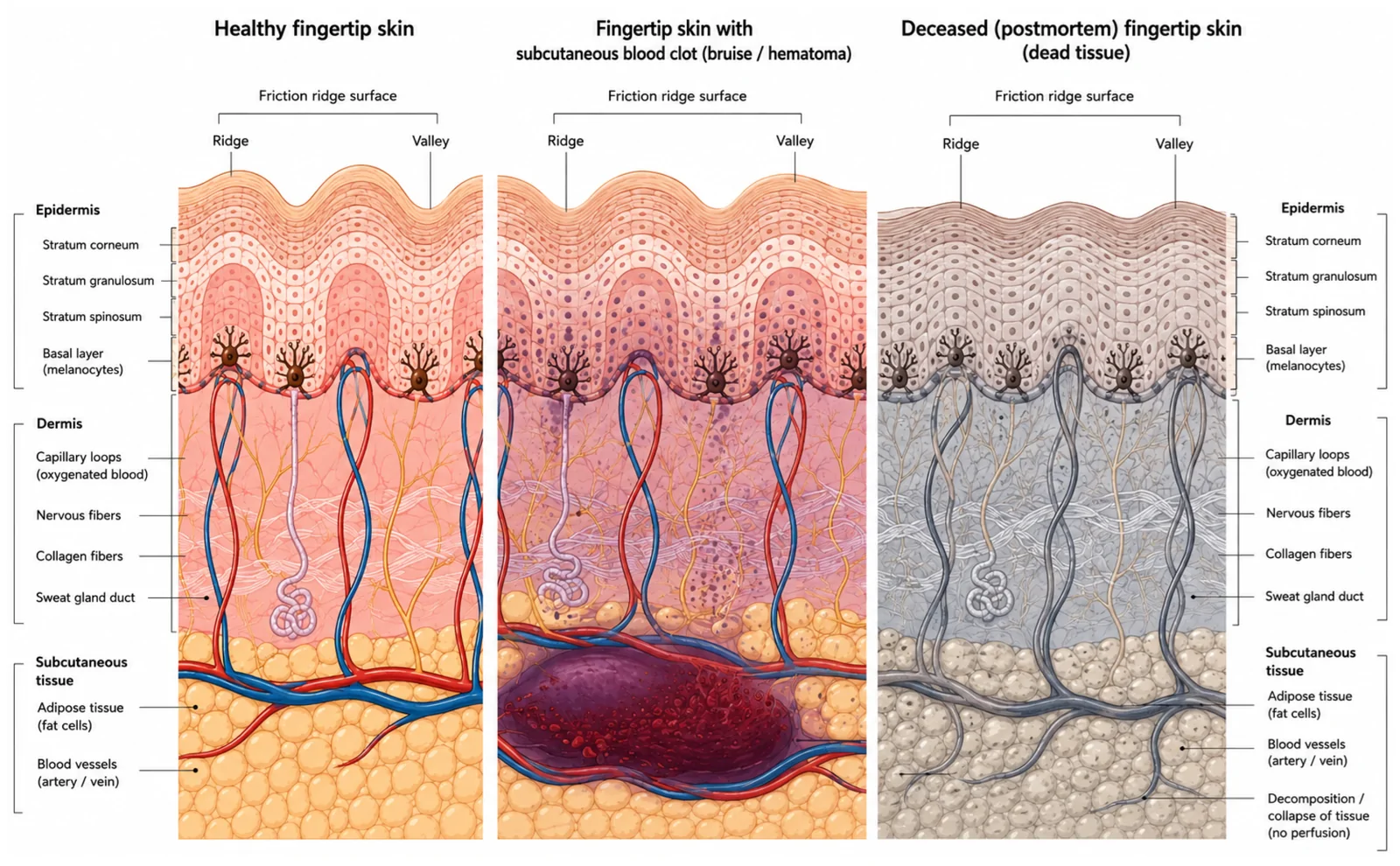
Cross-sectional comparison of healthy, hematoma-affected, and post-mortem fingertip skin.

Human skin consists primarily of the epidermis, dermis, and subcutaneous tissue, as schematically illustrated in Figure 1. For fingerprint acquisition, the epidermis is particularly important because its friction-ridge surface directly interacts with contact sensors and incident light. One relevant measurable property is *skin color* [13], determined predominantly by melanin [14] together with hemoglobin, other chromophores, and the optical properties of the surrounding tissue. Pigmentation affects wavelength-dependent absorption and scattering and is therefore particularly relevant to optical and camera-based sensing [15]. Local pathological or traumatic changes may further modify the observed color; for example, subcutaneous bleeding and hematoma formation can produce reddish, purple, or brown discoloration depending on the location and evolution of the affected tissue. In post-mortem subjects, the absence of active perfusion and subsequent tissue changes represent additional sources of optical variability. Consequently, measured color should be interpreted as the current optical appearance of the examined skin, rather than solely as an estimate of constitutive pigmentation.

In the proposed system, this optical response is represented by RGB components measured under defined illumination. Although RGB measurement does not provide full spectrophotometric characterization, it offers a compact quantitative descriptor suitable for comparison between measurement locations and with fingerprint acquisition results. This distinction is particularly relevant to the present post-mortem experiments, where measured RGB values may reflect not only baseline pigmentation but also local vascular, traumatic, and post-mortem changes.

A second key property is *skin moisture*. Excessively dry skin may result in insufficient ridge contact and discontinuous or low-contrast structures, whereas excessive moisture may reduce ridge–valley separation or cause neighboring structures to merge. The effect depends on the sensing principle and is particularly relevant to contact-based optical and capacitive sensors [3]. Moisture is also relevant to conventional forensic fingerprint recording, where the condition of the friction-ridge surface affects the transfer and visibility of the ridge pattern.

Additional parameters provide complementary information about the physiological state of the examined tissue. Surface temperature characterizes the local thermal state, while blood oxygenation can provide information about peripheral tissue perfusion in living subjects. Their interpretation is fundamentally different in post-mortem measurements, where active circulation and physiological oxygen delivery are absent. These parameters should therefore not be interpreted independently but together with color, galvanic skin response, and environmental conditions.

In the present work, skin condition is consequently treated as a *multidimensional state*. Skin color, galvanic skin response, surface temperature, and blood oxygenation, together with ambient illumination, temperature, and humidity, form the objective skin characterization subsequently related to fingerprint acquisition performance. This approach is particularly relevant to post-mortem fingerprint acquisition, where dehydration, loss of perfusion, local discoloration, tissue degradation, and other post-mortem changes can substantially alter the interaction between the finger and the acquisition system.

### 2.3. Fingerprint Image Quality Assessment

Fingerprint image quality is a key determinant of biometric recognition performance. A suitable image should contain a sufficiently large fingerprint area with distinguishable ridge–valley structures, adequate local contrast, and limited acquisition artefacts. Poor quality may lead to interrupted or merged ridges, incorrect minutiae extraction, and reduced matching performance [2]. Fingerprint quality should therefore be interpreted primarily as the *utility of an acquired sample for biometric recognition*, rather than as visual quality alone.

The most established objective quality assessment methods are the *NIST Fingerprint Image Quality* (NFIQ) algorithms. The original NFIQ was designed to predict fingerprint matcher performance from image characteristics [1]. Its successor, *NFIQ 2*, provides an open-source framework relating measurable image properties of 500 PPI optical and ink fingerprints to operational recognition performance [5,16]. Its quality measures are standardized within ISO/IEC 29794-4 [17], providing a reproducible basis for comparing fingerprint samples and acquisition conditions.

Commercial biometric systems additionally employ proprietary quality estimators integrated with their recognition pipelines. In the present work, licensed technologies from *Innovatrics* (https://www.innovatrics.com/, accessed on 3 September 2026) and *Neurotechnology* (https://www.neurotechnology.com/, accessed on 3 September 2026) are available for complementary quality evaluation. Innovatrics supports NFIQ/NFIQ 2-based quality assessment within its fingerprint acquisition solutions [18], while Neurotechnology VeriFinger [19] provides proprietary quality control capable of identifying acquisition problems such as excessively wet or dry fingers, inappropriate pressure, or incorrect placement, and also supports NFIQ 2.

Quality assessment is closely related to fingerprint image enhancement. Classical methods improve ridge visibility using normalization, local orientation and ridge-frequency estimation, and directional filtering [6]. More recent data-driven and AI-based methods offer further possibilities, but enhancement must preserve the original biometric information and must not introduce artificial minutiae or alter ridge topology.

In the proposed framework, fingerprint quality provides an objective feedback variable linking *skin condition, sensing technology, and acquisition outcome*. Insufficient quality may therefore trigger skin conditioning, repeated acquisition, selection of another sensing technology, or appropriate image enhancement. This establishes the basis for the skin-aware adaptive acquisition approach investigated in this work.

### 2.4. Artificial Intelligence in Fingerprint Acquisition and Preprocessing

Artificial intelligence and machine learning provide several opportunities to support fingerprint acquisition and processing. Data-driven methods can assist in *acquisition decision-making, fingerprint image-quality prediction, segmentation, ridge enhancement, orientation-field estimation, noise suppression, and restoration of degraded fingerprint images*. CNN-based approaches such as FingerNet and multi-task architectures have demonstrated that fingerprint processing can be learned jointly with fingerprint-specific structural information, particularly ridge orientation and minutiae [20,21]. U-Net-based approaches further demonstrate the potential of encoder–decoder architectures for enhancement and segmentation of low-quality fingerprint images [22].

Within the proposed skin-aware framework, AI and machine learning can be considered at two complementary stages: before and after fingerprint acquisition. Before acquisition, objectively measured skin and environmental parameters could serve as input features for supervised machine-learning models, such as random forests, gradient-boosted decision trees, or compact neural networks. Together with sensor-specific information and objective fingerprint image-quality measures such as NFIQ 2 [5], sufficiently large and appropriately labeled datasets could be used to train models for predicting expected acquisition quality and supporting decisions concerning skin conditioning or selection of a suitable sensing technology. Such functionality represents a proposed future extension of the present framework; no adaptive sensor-selection or acquisition decision-support model was trained or validated in the present study.

After acquisition, the captured fingerprint image and available contextual information could be used to guide adaptive image processing. Deep-learning methods have demonstrated the ability to enhance fingerprints acquired using different sensing technologies and to compensate for sensor-dependent noise and artefacts [23]. Within the proposed framework, measured skin properties could provide additional contextual information for selecting or parameterizing appropriate processing procedures. For example, fingerprints affected by excessive dryness and those affected by excessive moisture need not require identical enhancement strategies. This *context-aware enhancement* concept remains a proposed extension and was not implemented or quantitatively validated as an adaptive enhancement model in the present study.

An example of a modular framework combining conventional and learning-based fingerprint processing is the open-source pyfing library [24], which provides methods for fingerprint segmentation, orientation-field and ridge-frequency estimation, enhancement, and minutiae extraction. Its learning-based components include simplified U-Net segmentation and neural-network-based orientation, frequency, and enhancement methods. Although pyfing was not used as an adaptive skin-aware processing system in the present experiments, such modular implementations illustrate how individual preprocessing stages could in principle be selected or parameterized according to measured skin condition, sensing technology, and resulting fingerprint image quality.

Generative approaches, including GAN-based methods, have also been investigated for severely degraded fingerprint images. FingerGAN, for example, constrains latent fingerprint enhancement using fingerprint skeleton, minutiae, and orientation-field information [25]. Generative reconstruction, however, requires particular caution in biometric and forensic applications because a visually plausible fingerprint or an improved image-quality score does not necessarily demonstrate preservation of identity-bearing information. A generative model may synthesize ridge trajectories, endings, bifurcations, or connections that are not unambiguously supported by the acquired fingerprint. Consequently, improvement in fingerprint image quality must be distinguished from preservation of biometric fidelity. For forensic applications, processing should preferentially enhance information supported by the acquired image, such as ridge–valley separability, local contrast, illumination uniformity, and noise characteristics, while preserving ridge topology and minutiae. Any generatively reconstructed image should therefore be treated as a synthetic derived representation rather than as recovered original biometric evidence unless its forensic reliability has been independently established through appropriate biometric-fidelity validation.

In the present work, AI is therefore considered a potential supporting component of a future skin-aware acquisition pipeline rather than as an experimentally validated decision-making system. Multisensor measurements provide objective contextual information describing the physical condition of the skin and acquisition environment, while fingerprint-specific processing methods may subsequently improve the usability of the acquired image. Future integration of these components could enable data-driven support for skin conditioning, sensing-technology selection, reacquisition, and adaptive fingerprint processing according to the objectively measured skin condition, provided that such models are trained on sufficiently large and appropriately labeled datasets and independently validated for both fingerprint image quality and preservation of biometric identity.

### 2.5. Legal Aspects of Physiological and Biometric Data Acquisition

The legal framework governing the acquisition of *anatomical, physiological, and biometric characteristics* depends primarily on the purpose of the procedure and on whether it concerns a *living or deceased person*. Under Czech law, interference with the bodily integrity of a living person generally requires consent, unless authorized by law [26]. In criminal proceedings, the Code of Criminal Procedure permits, subject to statutory conditions, examination of the body, the taking of blood or other biological material, and procedures necessary for identification [27]. The Police Act likewise authorizes fingerprinting, recording of bodily characteristics, body measurements, imaging, and collection of biological samples in specifically defined situations [28].

Where such characteristics are processed as *personal data*, the applicable data-protection regime depends on the purpose of processing. Both the General Data Protection Regulation (GDPR) and Directive (EU) 2016/680 define *biometric data* as data resulting from specific technical processing of physical, physiological, or behavioral characteristics that allow or confirm unique identification, expressly including *dactyloscopic data* [29,30]. The GDPR provides the general EU framework, whereas processing by competent authorities for the prevention, investigation, detection, or prosecution of criminal offences falls within the law-enforcement regime of Directive (EU) 2016/680. *Physiological measurements are therefore not automatically biometric data*. The GDPR does not apply to personal data of deceased persons [29].

Czech law nevertheless protects the *human body after death*. The Civil Code permits an autopsy or other use of a deceased body without consent only where provided for by law [26]. The Health Services Act regulates autopsies and the scientific, research, or educational use of biological material; material obtained during healthcare or in connection with an autopsy may, under specified conditions, be used without consent if it is not linked to data enabling identification, subject to separate rules for genetic research [31]. In criminal proceedings, suspected criminal causation of death requires examination and autopsy of the body [27]. Comparable solutions exist abroad. German law permits certain identification measures on an accused person even against his or her will and expressly allows forensic identification measures and removal of cell tissue for molecular-genetic determination of the identity and sex of a deceased person [32]. In England, Wales, and Northern Ireland, the Human Tissue Act 2004 generally requires “*appropriate consent*” for specified uses of deceased bodies and human material, including research, subject to statutory exceptions [33].

## 3. Multisensor System for Objective Skin Property Assessment

The core of the proposed approach is a *portable and modular multisensor system* developed for objective and simultaneous assessment of physiological and optical skin properties together with relevant environmental conditions. The system is presented and experimentally applied in this work as a dedicated measurement platform for biometric acquisition, particularly fingerprint imaging. Its technical design is protected by Czech utility model CZ 39522 U1, *Multisensor Unit for Measuring Physiological Parameters of Human Skin* [4].

The system simultaneously measures *RGB skin color*, *non-contact surface temperature*, *galvanic skin response (GSR)* related to skin conductivity and moisture, and *blood oxygenation*. Ambient illumination, temperature, and relative humidity are recorded simultaneously to characterize the measurement environment. Together, these parameters provide a multidimensional description of the current skin condition that can subsequently be related to fingerprint acquisition and image quality.

**Figure 2:**
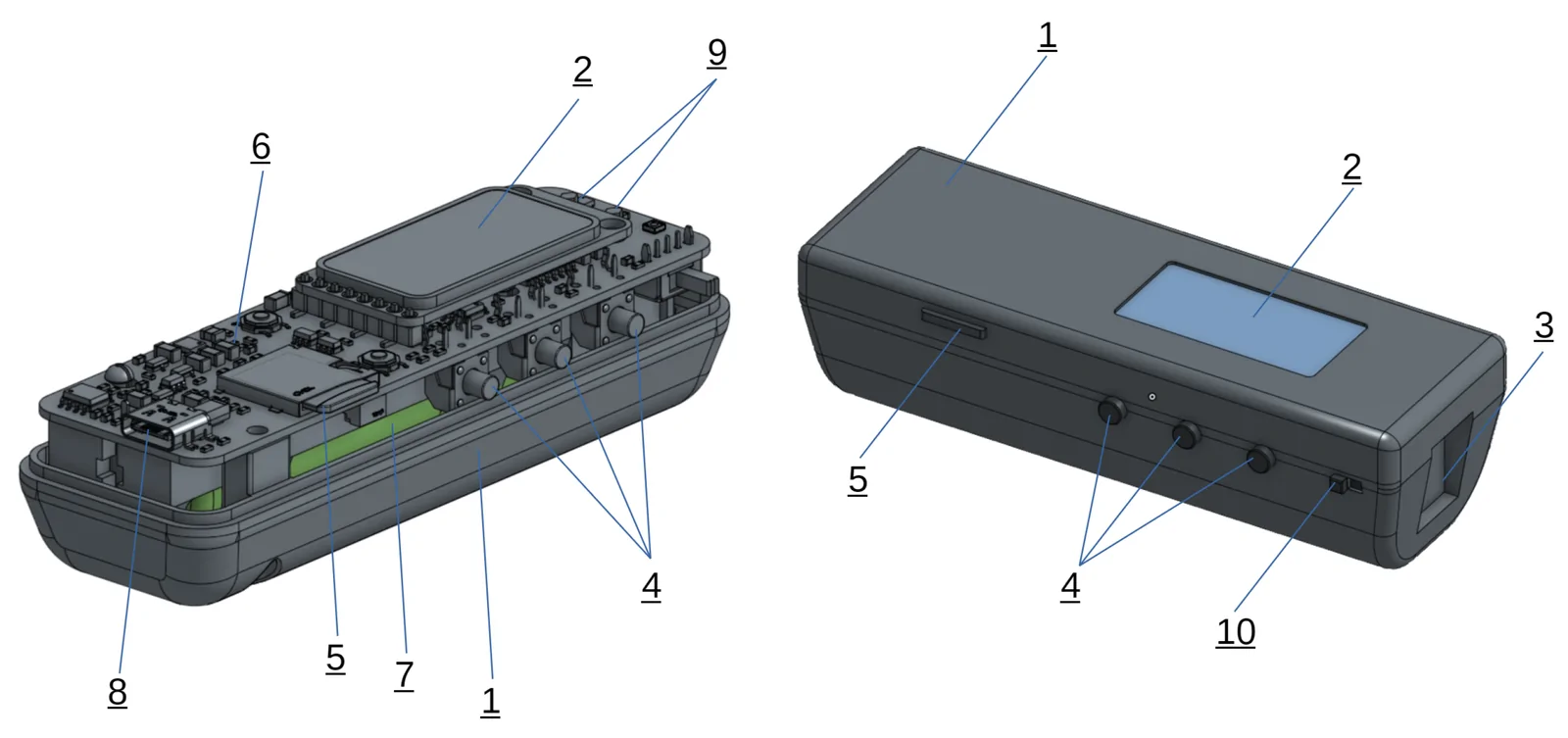
Technical drawing of the handheld control and data acquisition unit.

**Figure 3:**
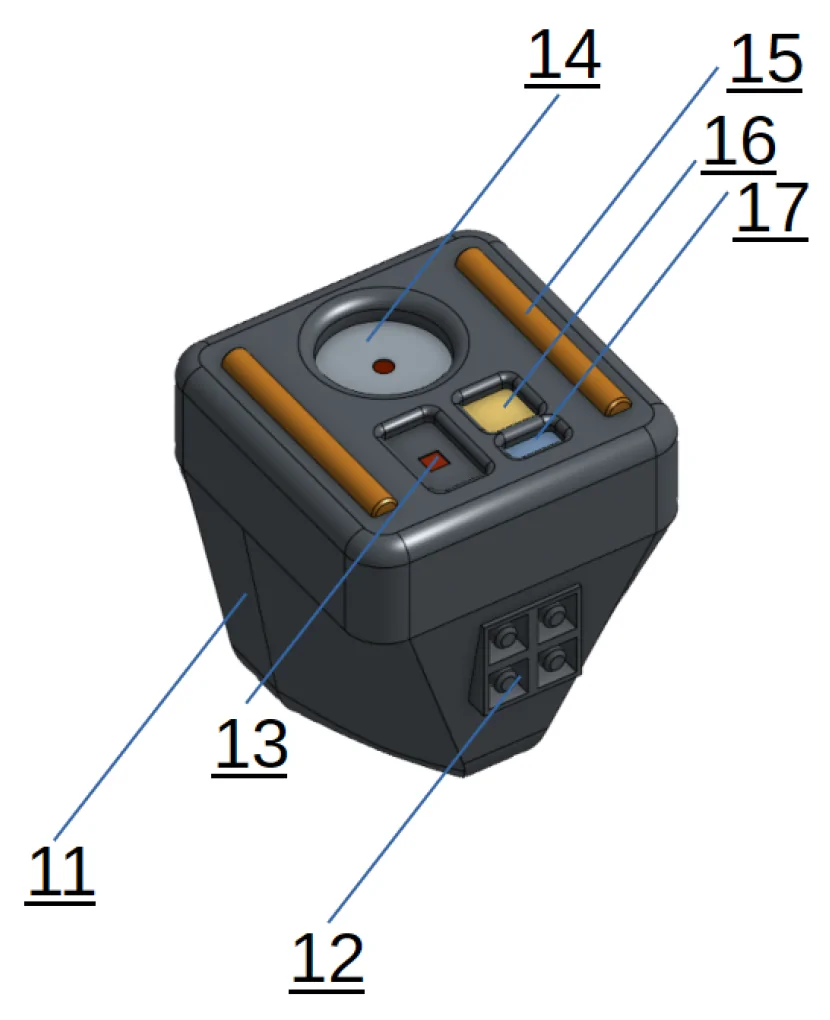
Technical drawing of the multisensor measurement head.

The system consists of two interconnected components: a *handheld control and data acquisition unit* (Figure 2) and a compact *multisensor measurement head* (Figure 3). The handheld unit integrates the control electronics, display, user controls, data storage, battery, and environmental sensors, whereas the measurement head contains the sensors interacting directly or optically with the examined skin. The two components are connected by cable, allowing the measurement head to be positioned independently on the selected skin area while the operator controls acquisition and data recording from the handheld unit.

Battery operation enables laboratory and field measurements without an external power source, which is particularly relevant to forensic applications. The modular architecture also allows different multisensor heads to be connected to the same handheld unit [4]. The developed measurement head combines several sensing principles within a compact surface, enabling multiple characteristics of the same local skin region to be acquired within a single measurement procedure.

### 3.1. Handheld Control and Data Acquisition Unit

The technical design and principal components of the handheld control and data acquisition unit are illustrated in Figure 2 and described below according to their numbered references. The handheld unit (1) provides control, acquisition, visualization, and storage functions for the multisensor system. The external components comprise a display (2), connector for the multisensor head (3), control buttons (4), memory-card slot (5), charging connector (8; wireless charging also possible), and power switch (10). Internally, the unit contains the main printed circuit board (6) and battery (7). A particularly important component for the proposed measurement methodology is the integrated set of sensors (9) for *ambient illumination, temperature, and relative humidity*, which records environmental conditions together with the physiological skin measurements [4].

The display (2) provides immediate feedback on the measured quantities, while the control buttons (4) allow the operator to navigate between measurement screens and control the acquisition procedure. The multisensor head is connected through connector (3), which provides communication with the measurement head and its power supply. The internal battery (7) enables portable operation and is charged through connector (8), typically implemented as USB, or using wireless charging; the design also permits optional wireless charging [4].

Acquired data are stored locally on a removable memory card using slot (5). Upon pressing the acquisition button, the measured values are recorded to the memory card in CSV format. Each measurement record is assigned a unique identifier and is accompanied by an acquisition timestamp obtained from the integrated real-time clock (RTC). These identifiers and timestamps provide unambiguous association of individual multisensor measurements with the corresponding experimental records and fingerprint samples acquired from the same examined subject. This linkage enables subsequent analysis of the relationship between *skin properties, environmental conditions, sensing technology, and fingerprint image quality*.

The environmental sensors (9) provide contextual information about the conditions under which each skin measurement is performed. Ambient temperature and relative humidity may influence perspiration and surface moisture, whereas ambient illumination is particularly relevant to RGB-based skin color assessment. These quantities are therefore recorded as *contextual variables rather than biometric characteristics*, improving the interpretation and reproducibility of measurements performed under different environmental conditions.

### 3.2. Multisensor Measurement Head

The technical design and principal sensing components of the multisensor measurement head are shown in Figure 3 and described below according to their numbered references. The multisensor head (11) is connected by cable to the handheld unit (1) through connector (3) on the handheld unit and connector (12) on the measurement head. According to the technical design, the head integrates an oximetric sensor (13) for blood oxygenation measurement, a non-contact infrared pyrometric sensor (14) for skin surface temperature measurement, a galvanic skin response (GSR) sensor (15) for skin moisture assessment, and a color sensor (16) with dedicated illumination (17) for controlled skin color measurement [4].

The sensing elements are integrated within a common compact housing, allowing several complementary properties of the same local skin region to be assessed without separate instruments. The measurement head is designed to be fully disinfectable, which enables repeated use and is particularly important for forensic and post-mortem examinations. The current functional prototype provides an *ingress protection* level approximately corresponding to IP44 and is intended primarily for experimental validation. The prototype and final production versions are planned with a fully sealed enclosure providing at least IP67 protection, ensuring complete protection against dust ingress and suitability for short water immersion under specified conditions. The head combines *contact and non-contact sensing principles*: GSR and oximetric measurements require interaction with the examined skin, whereas surface temperature is measured without contact and skin color is acquired optically under dedicated illumination. Together, RGB color, GSR, surface temperature, and blood oxygenation provide a *multidimensional characterization of the current skin condition* rather than a simple categorical distinction between *dry*, *normal*, and *moist* skin. The modular architecture additionally allows alternative measurement heads to be connected to the handheld unit [4].

### 3.3. Measured Skin Parameters

The multisensor head acquires four complementary skin parameters: *RGB color, surface temperature, galvanic skin response (GSR), and blood oxygenation*. These measurements characterize different optical and physiological properties of the examined skin and provide quantitative descriptors that can subsequently be related to fingerprint acquisition and image quality [4].

#### 3.3.1. Skin Color

Skin color is acquired using an optical color sensor with dedicated illumination and is represented by calibrated RGB values. To reduce sensor-dependent deviations, the raw responses $r={\left[R_{\mathrm{raw}},G_{\mathrm{raw}},B_{\mathrm{raw}}\right]}^{T}$ are transformed using a calibrated 3×4 color correction matrix (CCM),

(1) $$\left[\begin{array}{c} R_{\mathrm{cal}}\\ G_{\mathrm{cal}}\\ B_{\mathrm{cal}} \end{array}\right]=\left[\begin{array}{cccc} m_{11} & m_{12} & m_{13} & b_{1}\\ m_{21} & m_{22} & m_{23} & b_{2}\\ m_{31} & m_{32} & m_{33} & b_{3} \end{array}\right]\left[\begin{array}{c} R_{\mathrm{raw}}\\ G_{\mathrm{raw}}\\ B_{\mathrm{raw}}\\ 1 \end{array}\right],$$

where $m_{ij}$ are the color correction coefficients and $b_{i}$ are channel-specific offsets. The calibration matrix is derived from measurements of known reference colors using weighted least-squares estimation. An inverse-luminance weighting is applied during calibration to increase the contribution of darker reference colors and reduce the dominance of bright samples in the fitting process. At least four calibration points are required, while the implemented calibration dataset contains 13 reference colors covering dark, neutral, and chromatic regions.

After application of the CCM, the compensated RGB components are normalized to the interval [0,1]. No gamma correction is applied to the values used for quantitative analysis. The resulting compensated skin color is expressed as

(2) $$c*\mathrm{RGB}={\left[\begin{array}{ccc} R*\mathrm{comp} & G_{\mathrm{comp}} & B_{\mathrm{comp}} \end{array}\right]}^{T},\;R_{\mathrm{comp}},G_{\mathrm{comp}},B_{\mathrm{comp}}\in \left[0,1\right].$$

For visualization, the normalized components can subsequently be converted to an 8-bit RGB representation by multiplication by 255.

Dedicated illumination together with color calibration improves measurement consistency and reduces variability associated with the sensor response and uncontrolled ambient lighting. The resulting RGB values provide a quantitative descriptor of the optical appearance of the examined skin and can subsequently be related to fingerprint image quality. Temporary factors affecting skin appearance, including tanning, hematoma, surface contamination, or other local pigmentation changes, should nevertheless be considered when interpreting the measured values.

#### 3.3.2. Skin Surface Temperature

Skin surface temperature was measured using a Melexis MLX90614ESF-BAA non-contact infrared thermopile sensor. The sensor is factory calibrated over an object-temperature range of −70 to 380 °C and an ambient sensor-temperature range of −40 to 125 °C. The digital SMBus output provides a temperature resolution of 0.02 °C. According to the manufacturer, an accuracy of approximately ±0.5 °C is specified for both ambient and object temperatures within the 0 to 50 °C range. The sensor was operated using the factory-default emissivity setting of ε=1.0.

The very low temperatures observed in several post-mortem cases fall within the calibrated object-temperature range of the sensor, but below the temperature interval for which the manufacturer specifies the standard ±0.5 °C accuracy. These measurements should therefore be interpreted with increased uncertainty. In particular, the minimum values reported in Table 1 reflect measurements obtained from refrigerated post-mortem tissue and should not be interpreted as having the same measurement accuracy as values obtained within the manufacturer’s standard accuracy range.

The measured skin surface temperature provides complementary information about the physiological and thermal condition of the examined region. In addition, ambient temperature is recorded independently by the environmental sensing subsystem of the handheld unit. The availability of *skin surface, sensor-head, and ambient temperatures* allows the measured skin temperature to be interpreted in the context of both the sensing system and surrounding environmental conditions.

#### 3.3.3. Galvanic Skin Response and Moisture

Skin moisture is assessed through the electrical response of the skin using the *galvanic skin response* (GSR) sensing circuit. In the implemented system, the measured voltage is converted into an estimate of skin resistance using the voltage-divider relationship

(3) $$R_{\mathrm{skin}}=\frac{V_{\mathrm{GSR}}R_{\mathrm{ref}}}{V_{\mathrm{ref}}-V_{\mathrm{GSR}}},$$

where $V_{\mathrm{GSR}}$ is the reconstructed GSR input voltage, $V_{\mathrm{ref}}$ is the adjustable reference voltage of the GSR circuit, and $R_{\mathrm{ref}}=1\;k\Omega$ is the known resistance of the measurement circuit. The reference voltage is set using an on-board potentiometer and its actual value is measured through a dedicated ADC channel. Consequently, the measured reference voltage, rather than a fixed nominal voltage, is used in the calculation of $R_{\mathrm{skin}}$. The resulting resistance provides a quantitative electrical descriptor related to the current moisture condition of the examined skin.

This parameter is particularly relevant to fingerprint acquisition because surface moisture affects the interaction between papillary ridges and contact-based sensing surfaces. Instead of assigning the skin subjectively to *dry*, *normal*, or *moist* categories, the developed system records a numerical value that can subsequently be associated with fingerprint image quality and compared across different sensing technologies.

#### 3.3.4. Blood Oxygenation and Photoplethysmography

The multisensor head incorporates an optical oximetric sensor based on photoplethysmographic (PPG) sensing. In addition to blood oxygenation assessment, the implemented acquisition system processes the infrared PPG signal to obtain information on peripheral pulsation. The IR samples are accumulated in a circular buffer and analyzed by a peak-detection algorithm. Candidate pulse peaks are identified relative to the signal mean and standard deviation, filtered according to their prominence and minimum temporal separation, and the heart rate is subsequently calculated from the average peak-to-peak interval,

(4) $$HR=\frac{60f_{s}}{\bar{\Delta n}},$$

where HR is the heart rate in beats per minute (BPM), $f_{s}$ is the sampling frequency, and $\bar{\Delta n}$ is the average distance between detected pulse peaks expressed in samples. The implementation requires at least 100 samples and accepts calculated heart-rate values within the physiological range of 30–200 BPM.

Oximetric and PPG measurements provide complementary information about the physiological state and perfusion of the examined tissue. Unlike skin resistance or RGB color, these parameters are not assumed to directly determine fingerprint image formation. Their inclusion provides a broader characterization of tissue condition and enables investigation of whether physiological state, including the presence or absence of detectable peripheral pulsation, is associated with fingerprint acquisition quality. This information is particularly relevant to the post-mortem measurements considered in this work.

This measurement is naturally of limited relevance to post-mortem fingerprint acquisition, where active peripheral perfusion and pulsatile blood flow are absent. In living subjects, however, PPG and oximetric information may provide useful complementary context for interpreting the physiological state of the examined finger. In particular, reduced peripheral perfusion may be associated with colder extremities; therefore, combining PPG-derived information with skin surface temperature may help distinguish whether a low finger temperature reflects environmental conditions or a physiological reduction in peripheral blood flow.

The PPG, blood-oxygenation, and heart-rate estimation functions described above represent capabilities of the developed multisensor platform intended primarily for measurements in living subjects. These modalities were not evaluated in the present post-mortem experiment because all examined subjects were deceased and therefore lacked active peripheral circulation. Consequently, PPG-derived parameters did not contribute to the experimental analyses or conclusions reported in the post-mortem experimental analysis.

### 3.4. Synchronized Measurement and Data Flow

A key feature of the developed system is the synchronized acquisition of skin-related and environmental parameters. During each measurement, the multisensor head acquires optical and physiological skin data, while the handheld unit simultaneously records the corresponding environmental conditions. The acquired values are processed by the control electronics, displayed to the operator, and stored for subsequent analysis. The resulting measurement can be represented by the feature vector

(5) $$x_{\mathrm{meas}}={\left[R,\;G,\;B,\;T_{\mathrm{skin}},\;R_{\mathrm{skin}},\;O_{\mathrm{PPG}},\;E_{\mathrm{amb}},\;T_{\mathrm{amb}},\;RH_{\mathrm{amb}}\right]}^{T},$$

where *R*, *G*, and *B* are the calibrated skin color components, $T_{\mathrm{skin}}$ is the non-contact skin surface temperature, $R_{\mathrm{skin}}$ represents the skin resistance derived from the GSR measurement, and $O_{\mathrm{PPG}}$ denotes the oximetric/photoplethysmographic measurement output. The environmental variables $E_{\mathrm{amb}}$, $T_{\mathrm{amb}}$, and $RH_{\mathrm{amb}}$ represent ambient illumination, temperature, and relative humidity, respectively.

This feature vector provides a unified quantitative description of the measured skin state and its acquisition environment. Individual quantities may subsequently be normalized or transformed, and additional derived features can be introduced before statistical or machine-learning analysis. Most importantly, the stored measurement can be associated with the corresponding fingerprint samples, enabling relationships between skin condition, environmental conditions, sensing technology, and resulting fingerprint image quality to be investigated.

### 3.5. Relevance to Fingerprint Acquisition

The purpose of the developed system is to provide an *objective description of skin condition before fingerprint acquisition*. Conventional fingerprint capture primarily evaluates the resulting biometric sample, whereas information about the physiological and optical state of the finger at the time of acquisition is generally unavailable. Consequently, reduced fingerprint quality can be detected after capture, but its relationship to the actual skin condition cannot be directly assessed.

By associating each fingerprint sample with the simultaneously measured skin and environmental parameters, the proposed approach enables the relationship

(6) $$Q=f\left(x_{\mathrm{meas}},s\right),$$

to be investigated, where *Q* denotes fingerprint image quality, $x_{\mathrm{meas}}$ is the measured skin and environmental feature vector defined in Equation (5), and *s* represents the fingerprint sensing technology.

The central hypothesis is that the relationship between skin condition and fingerprint quality is *sensor dependent*; the same skin state may affect different acquisition principles differently. Pre-acquisition skin assessment may therefore support appropriate skin conditioning, sensor selection, or adaptation of acquisition parameters. The measured context can also be retained for subsequent quality assessment and image enhancement, including data-driven and AI-based methods. The developed multisensor system thus provides the measurement basis for the skin-aware fingerprint acquisition framework evaluated in the following sections.

## 4. Experimental Measurements and Results

The experimental study was conducted under real forensic conditions at the Institute of Forensic Medicine, University Hospital Královské Vinohrady, Prague, Czech Republic. A total of *50 deceased subjects* representing various post-mortem intervals, causes of death, and skin conditions were examined. For each subject, the experimental protocol was restricted to the *right hand*: fingerprints from all five fingers were acquired using the evaluated fingerprint sensing technologies, while skin and environmental parameters were recorded using the developed multisensor system.

Multisensor measurements were performed at two standardized anatomical locations: the *middle phalanx of the right middle finger* and the *inguinal region*. The measurement protocol included calibrated RGB skin color, non-contact surface temperature, GSR-derived skin resistance, and ambient illumination, temperature, and relative humidity. Although the developed multisensor platform also supports PPG-based blood-oxygenation and heart-rate measurements, these modalities were not evaluated in the present experiment because the study population consisted exclusively of deceased subjects without active peripheral circulation. The post-mortem analysis therefore focused on sensing modalities that remained physically meaningful under the experimental conditions. PPG-derived variables were excluded from all subsequent analyses and did not contribute to the results or conclusions reported for the post-mortem dataset.

Numerical multisensor records were available for *47 of the 50 examined subjects*. The remaining three subjects were included in the fingerprint acquisition experiment but were excluded from quantitative analyses requiring the corresponding multisensor data. Within the group of 47 subjects with available multisensor records, finger skin resistance was successfully measured in 43 cases; in the remaining four cases, GSR measurement could not be performed due to the condition of the post-mortem skin.

### 4.1. Skin Measurement Protocol

A standardized measurement protocol was applied to all examined subjects. Skin measurements were performed at two anatomical locations: the *middle phalanx of the right middle finger* and the *inguinal region*. The middle phalanx was selected as a representative site close to the fingerprint-bearing distal phalanx while providing sufficient area for stable placement of the multisensor head without interfering with subsequent fingerprint acquisition.

The inguinal region served as a reference site for skin color assessment because it is typically less affected by solar exposure and tanning than the hands. Comparison of the finger and inguinal measurements therefore provides complementary information for distinguishing general skin pigmentation from local optical changes in the hand.

At both locations, the system recorded calibrated RGB skin color, non-contact surface temperature, and skin resistance derived from the GSR measurement. Ambient illumination, temperature, and relative humidity were recorded simultaneously. Oximetric and PPG parameters were excluded from the post-mortem protocol because active peripheral perfusion was absent.

**Figure 4:**
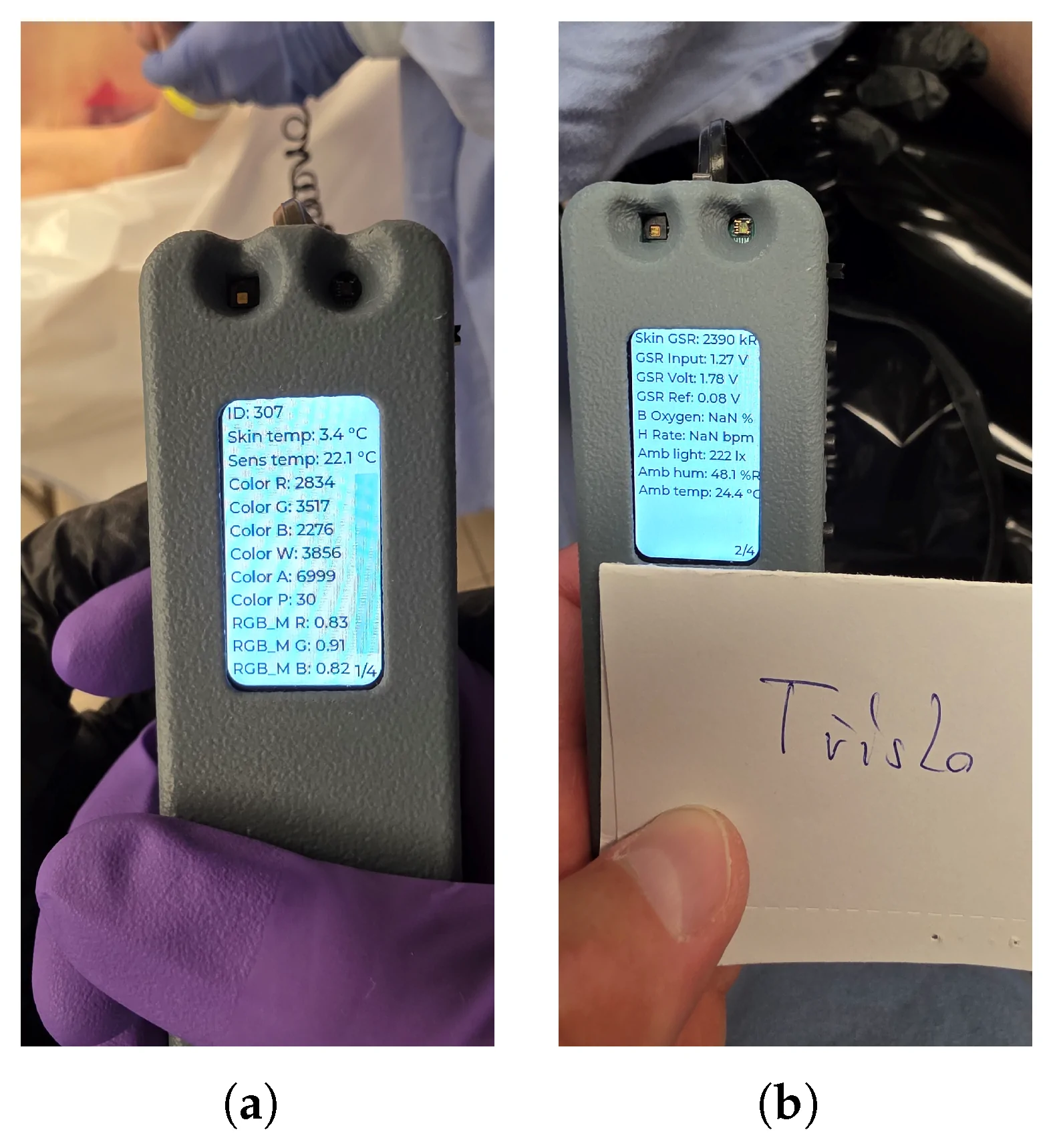
Illustrative post-mortem skin measurement values displayed on the device screen for (**a**) the middle phalanx of the right middle finger and (**b**) the inguinal reference region.

Figure 4 shows representative measurements obtained during the forensic experiment. Figure 4a demonstrates measurement at the middle phalanx of the right middle finger, with the device displaying skin temperature and color-related parameters. Figure 4b shows measurement at the inguinal reference site, including skin resistance and the simultaneously recorded environmental conditions. The displayed values also provided immediate feedback to the operator during examination.

### 4.2. Environmental and Skin Measurement Conditions

Numerical multisensor data were available for 47 of the 50 examined subjects. The measurements were performed under varying but recorded environmental conditions, with ambient temperature ranging from 20.4 to 26.5 °C and relative humidity from 29.0% to 64.0%. The maximum relative humidity of 64.0% was recorded during forensic preparation involving the boiling of a skull, which generated a substantial amount of water vapor in the examination room. These contextual variables were recorded because environmental conditions may affect the measured state of the skin and are therefore relevant to the interpretation and reproducibility of the post-mortem measurements.

The principal quantitative parameters are summarized in Table 1.

Substantial inter-subject variability was observed, particularly in finger skin resistance, which ranged from 46 to 3294 kΩ (median 705 kΩ). Because the GSR-based measurement reflects the electrical properties of the skin and is sensitive to its moisture state, this wide range indicates markedly different post-mortem skin conditions among the examined subjects. In general, lower resistance corresponds to a more conductive skin interface, whereas higher resistance is consistent with a less conductive, typically drier surface. This variability was also consistent with the practical conditions encountered during forensic examinations. Some subjects were measured shortly after autopsy and washing, when the skin surface could remain relatively moist, whereas others were examined immediately after removal from refrigerated post-mortem storage, where prolonged cooling under low-humidity conditions can result in a substantially drier skin surface. Additional variability may have resulted from measurement-related factors, particularly small differences in the positioning and contact of the GSR sensor at the examined site. This effect may have been more pronounced in the inguinal region, where local hair can interfere with uniform sensor–skin contact and thereby contribute to fluctuations in the measured resistance. These physiological, environmental, and measurement-related factors provide a plausible explanation for part of the observed variation in skin resistance and further emphasize the importance of objective skin assessment immediately before fingerprint acquisition.

Skin temperature also varied considerably, ranging from −11.7 to 30.3 °C at the finger and from −2.0 to 27.1 °C at the inguinal site. The wide temperature range reflects the heterogeneous thermal conditions encountered in routine post-mortem practice. Subjects examined shortly after removal from refrigerated storage could exhibit very low, occasionally sub-zero, peripheral skin temperatures, particularly at the fingers. In contrast, subjects that had remained in the examination room for a longer period showed progressive thermal equilibration with the surrounding environment. Higher skin temperatures could additionally occur under temporarily elevated room temperature and humidity; for example, during forensic procedures generating substantial amounts of steam. These observations emphasize that post-mortem skin temperature is strongly dependent on the conditions preceding acquisition and should therefore be interpreted together with ambient temperature and other contextual variables. Combined with skin resistance and RGB color, these measurements provide a quantitative description of the actual tissue state at the time of fingerprint acquisition.

### 4.3. Distribution of Finger Skin Resistance

**Figure 5:**
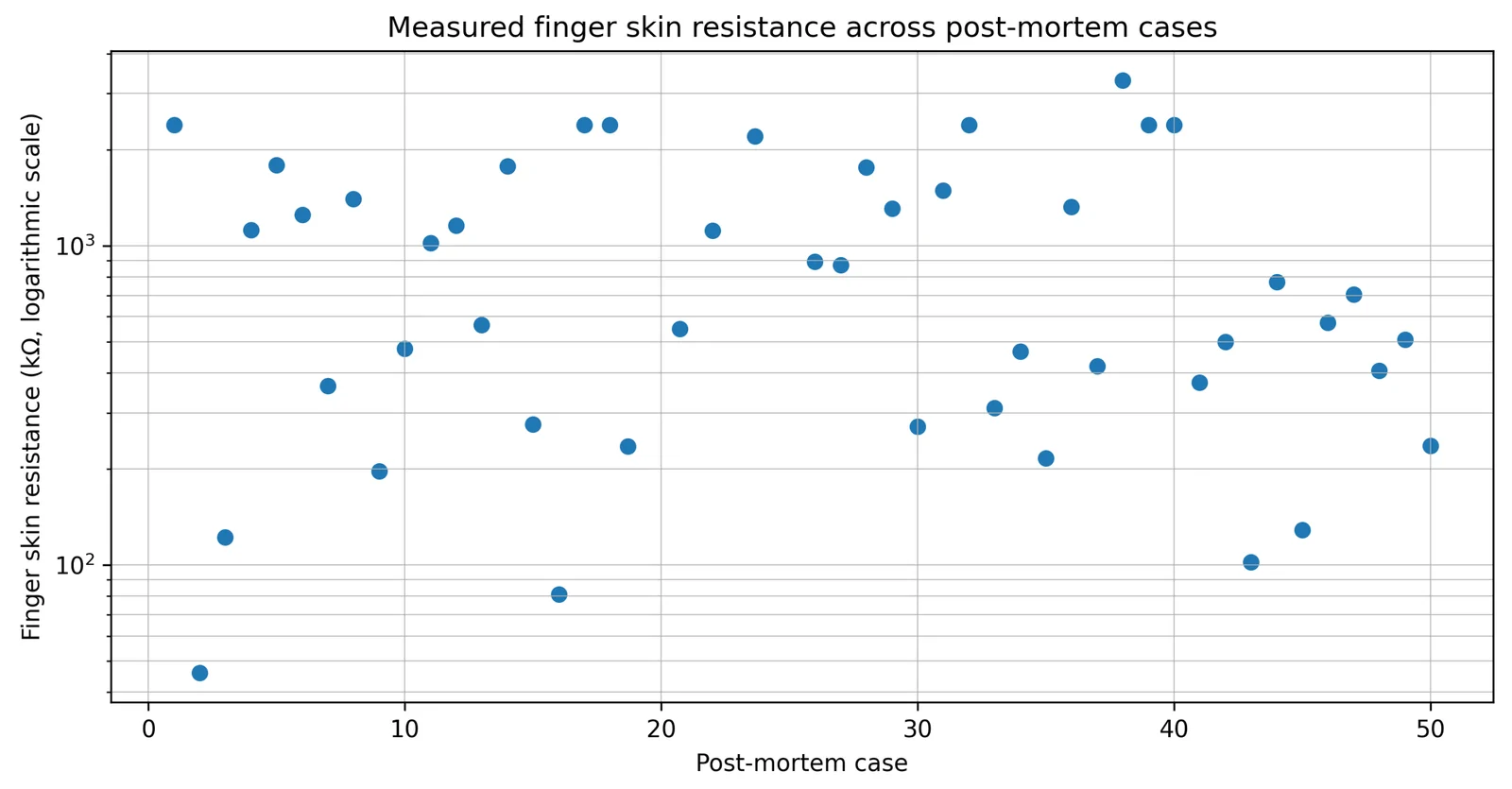
Distribution of finger skin resistance in 43 post-mortem cases measured at the middle phalanx of the right middle finger.

Figure 5 shows the individual skin resistance values measured at the middle phalanx of the right middle finger for the 43 cases in which GSR measurement was successfully performed. The values ranged from 46 to 3294 kΩ and exhibited a strongly dispersed distribution; a logarithmic vertical scale is therefore used to visualize both low- and high-resistance measurements.

The coefficient of variation was approximately 85%, demonstrating substantial inter-subject variability in the electrical condition of the post-mortem skin. As discussed above, this variability is consistent with the heterogeneous conditions encountered during forensic examination, including recently washed subjects and subjects examined shortly after removal from refrigerated storage. Such differences are particularly relevant to contact-based fingerprint acquisition, for which markedly different skin conditions may alter the finger–sensor interaction.

The measured resistance should not be interpreted as a direct measurement of tissue water content. It represents an electrical property of the skin obtained using the GSR-based measurement circuit and is used here as a quantitative indicator related to the current moisture condition of the examined skin. Lower resistance generally corresponds to a more conductive and typically more moist skin interface, whereas higher resistance is consistent with a less conductive and typically drier surface. This objective measurement provides a substantially more reproducible descriptor than a purely visual or tactile classification of the finger as *dry*, *normal*, or *moist*.

### 4.4. Post-Mortem Skin Color

Skin color measurements showed considerable inter-subject variability and differences between the finger and the inguinal reference site. For each measurement, both raw sensor responses and compensated RGB components were recorded. The compensated components are stored as normalized values in the interval [0,1] and can therefore be converted to an 8-bit RGB representation as

(7) $$c_{8\mathrm{bit}}=255{\left[\begin{array}{ccc} R_{\mathrm{comp}} & G_{\mathrm{comp}} & B_{\mathrm{comp}} \end{array}\right]}^{T}.$$

This representation enables intuitive reconstruction of the measured skin color while preserving the calibrated sensor output for quantitative analysis. The inguinal region was used as a within-subject reference because it is generally less affected by solar exposure, tanning, and occupational or environmental influences than the hand. Consequently, differences between the two anatomical sites may provide additional information beyond the absolute RGB values.

Because both measurements are obtained from the same individual, the within-subject comparison partially controls for inter-individual differences in constitutive pigmentation and emphasizes local optical alterations of the hand associated with environmental exposure, trauma, or post-mortem change.

Post-mortem skin color should not be interpreted as a measure of pigmentation alone. In addition to melanin-related pigmentation, the measured optical appearance may reflect absent perfusion, post-mortem redistribution of blood, local discoloration, hematoma, and other tissue changes. In the present study, calibrated RGB values are therefore treated as quantitative optical descriptors of the skin state at the time of fingerprint acquisition.

To characterize local differences while partially reducing the influence of general subject-specific pigmentation, a within-subject RGB difference vector was defined as

(8) $$\Delta c_{\mathrm{RGB}}=c_{\mathrm{finger}}-c_{\mathrm{inguinal}}.$$

Table 2 summarizes the compensated RGB measurements and the corresponding within-subject differences for the 47 cases with complete multisensor records.

The median compensated finger color was (R,G,B)=(0.810,0.790,0.730), compared with (0.827,0.820,0.730) at the inguinal reference site. The corresponding median within-subject differences were small (ΔR=−0.020, ΔG=−0.030, and ΔB=0.010), indicating no pronounced systematic color shift between the two anatomical sites at the group level. Individual differences were considerably larger, however, ranging from −0.178 to 0.190 for ΔR, from −0.240 to 0.240 for ΔG, and from −0.185 to 0.230 for ΔB. This substantial subject-specific variability suggests that within-subject color differences may provide a more informative descriptor of local post-mortem skin appearance than absolute RGB values alone. These descriptors can subsequently be evaluated together with skin resistance, temperature, environmental conditions, fingerprint sensing technology, and resulting image quality.

### 4.5. Fingerprint Acquisition and Skin Conditioning

Following multisensor skin assessment, fingerprints from all five fingers of the right hand were acquired using several technologically different systems. The experimental setup included a contactless fingerprint acquisition system from **IDLoop** (https://www.idloop.com/, accessed on 3 September 2026), commercial **optical DY50** (https://www.laskakit.cz/en/snimac-otisku-prstu-s-pameti-dy50/, accessed on 3 September 2026) and **capacitive FPC1020AM** (https://www.laskakit.cz/en/kapacitni-snimac-otisku-prstu-fpc1020am-s-pameti-500-otisku/, accessed on 3 September 2026) sensors, **Jenetric LiveTouch Duo** (https://www.jenetric.com/en/products/livetouch-duo.html, accessed on 3 September 2026) acquisition device, and an experimental fingerprint acquisition system **TBS 3D FLOW** (the TBS 3D FLOW system was developed within the project “*Multimodal biometric device for verifying the identity of persons based on fingerprints and faces when crossing state borders (BEFFIC)*”, supported by the Ministry of the Interior of the Czech Republic, project No. VB01000066, 2022–2023). In addition, conventional inked fingerprint (dactyloscopic) cards were produced where applicable as a *forensic reference*. The use of various acquisition principles enabled the same post-mortem hand to be evaluated under comparable skin conditions while exposing differences in the interaction between the friction-ridge surface and individual sensing technologies.

A practically important observation concerned excessively dry and excessively moist fingers. Both conditions were encountered during the post-mortem examinations and could substantially impair acquisition. Excess surface moisture was removed using cellulose tissue, whereas excessively dry skin was temporarily moistened using a disinfectant wipe. Fingerprint acquisition was subsequently repeated. In a number of initially problematic cases, this simple conditioning visibly improved acquisition across the tested sensing technologies, indicating that poor initial image quality did not necessarily result from deterioration of the friction-ridge structure itself, but could arise from an unfavorable instantaneous condition of the skin–sensor interface.

This observation is particularly relevant to the GSR-based skin-resistance measurement. In conventional forensic practice, the decision to dry or moisten a finger is largely based on visual inspection, touch, and operator experience. The developed multisensor system provides an additional quantitative descriptor of the skin condition before acquisition and therefore creates the possibility of replacing or complementing subjective assessment with a reproducible decision criterion.

The resulting acquisition strategy can be expressed conceptually as

(9) $$\left(a^{*},s^{*}\right)=\arg\max_{a,s}P\left(Q_{a,s}\geq Q_{\min}|x_{\mathrm{meas}}\right),$$

where $x_{\mathrm{meas}}$ represents the measured skin and environmental parameters, *a* denotes a possible pre-acquisition action (*no intervention*, *drying*, or *moistening*), *s* represents the fingerprint sensing technology, $Q_{a,s}$ is the resulting fingerprint image quality, and $Q_{\min}$ denotes the required quality threshold.

The proposed strategy therefore extends simple sensor selection to a combined *skin-conditioning and sensor-selection problem*. Depending on the measured skin state, the preferred action may be to acquire the fingerprint directly, condition the skin before acquisition, select a different sensing technology, or combine these measures. With a sufficiently large annotated dataset, the relationship between $x_{\mathrm{skin}}$, conditioning action, sensing technology, and resulting image quality could subsequently be learned using statistical or machine-learning models.

### 4.6. Practical Findings and Implications

The post-mortem experiment demonstrated substantial variability in the condition of fingerprint-bearing skin. The wide range of measured skin resistance, together with the practical need to dry or moisten individual fingers before repeated acquisition, confirms that post-mortem skin cannot be considered a constant interface between the finger and the sensor. Objective assessment immediately before acquisition can therefore provide useful information that is otherwise based primarily on visual inspection, touch, and operator experience.

**Figure 6:**
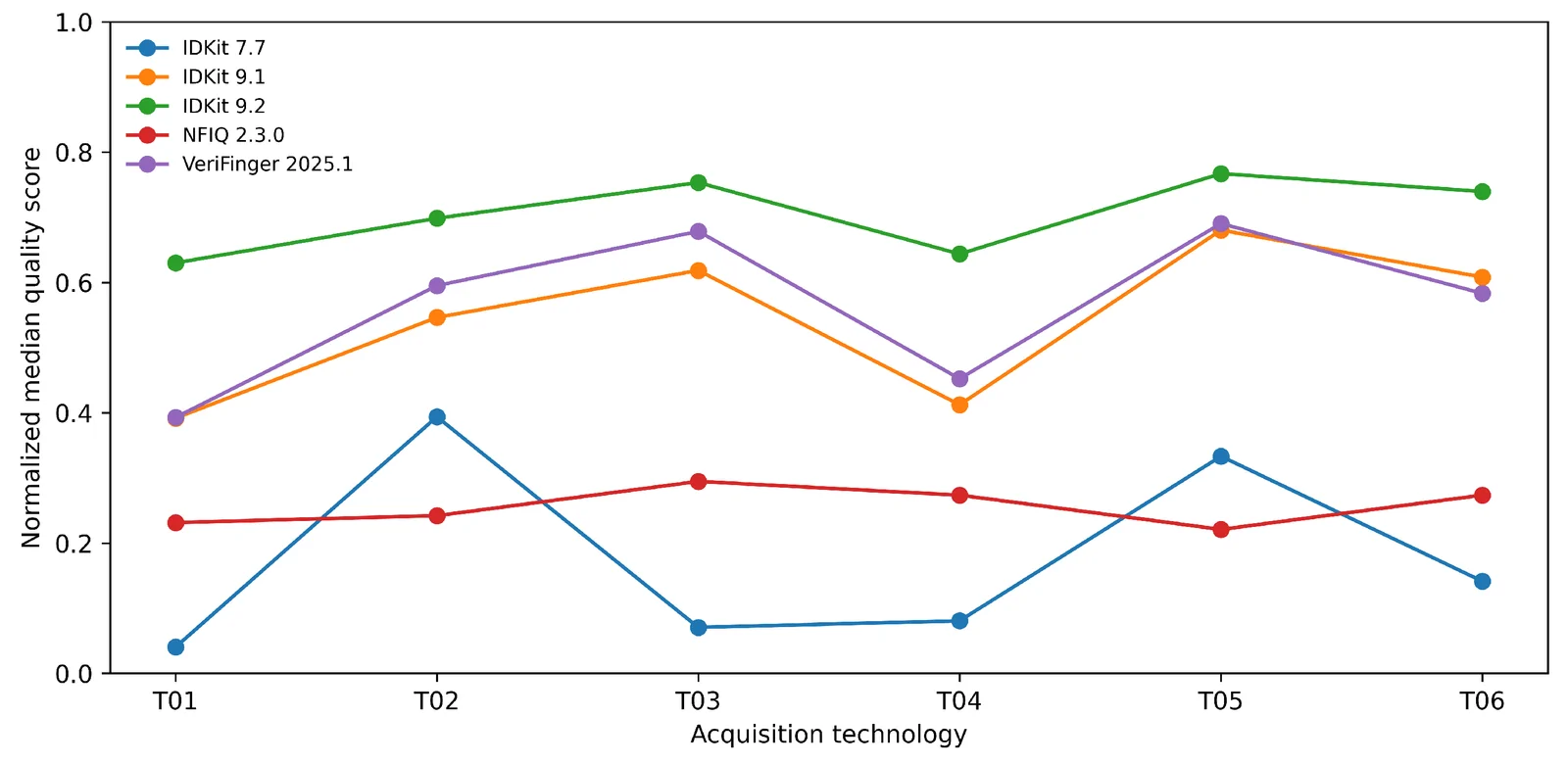
Median normalized fingerprint image-quality scores obtained using the evaluated acquisition technologies. Quality scores were normalized to the interval [0,1], with higher values consistently representing better fingerprint image quality.

The acquired fingerprint images were subsequently evaluated using the available image-quality measures, and the resulting scores were normalized to enable comparison between acquisition technologies with different quality-score ranges. Figure 6 summarizes the median normalized quality values obtained for the evaluated sensing technologies.

The acquired fingerprint images were subsequently evaluated using the available image-quality measures. Because the evaluated acquisition technologies provided quality scores on different native numerical scales, the scores were transformed to a common interval [0,1] using min–max normalization, defined as $Q_{\mathrm{norm}}=\left(Q-Q_{\min}\right)/\left(Q_{\max}-Q_{\min}\right)$, where *Q* denotes the original quality score and $Q_{\min}$ and $Q_{\max}$ denote the lower and upper limits of the corresponding native quality-score scale. For all quality measures included in this comparison, higher native scores represented better fingerprint image quality; therefore, no inversion of score direction was required. Consequently, higher values of $Q_{\mathrm{norm}}$ consistently represent better fingerprint image quality across all evaluated acquisition technologies. Figure 6 summarizes the median normalized quality values obtained for the evaluated sensing technologies.

A particularly important practical observation was that reduced fingerprint quality was frequently associated with pronounced morphological alterations of the post-mortem friction-ridge skin, including *wrinkling, folding, ridge flattening*, and *geometrical deformation of the ridge-bearing surface*. Such alterations can impair ridge continuity, local ridge orientation, ridge–valley contrast, and the subsequent extraction of identity-bearing biometric features. However, this type of morphological degradation is not directly represented by the currently measured RGB skin color, GSR-derived skin resistance, or surface temperature. A finger may therefore exhibit values of these parameters within otherwise unremarkable ranges while still producing a poor-quality fingerprint because the geometry of the friction-ridge surface has deteriorated.

These observations indicate that a skin-aware acquisition strategy should not rely on any single measured skin parameter. Instead, optical, electrical, thermal, and environmental measurements should ultimately be considered together with information describing the *morphological condition of the friction-ridge surface* and with objective fingerprint image-quality measures. In practical use, such combined information could support decisions concerning direct acquisition, controlled drying or moistening of the skin, selection of an alternative sensing technology, reacquisition, or subsequent conservative preprocessing.

The present analysis does not assign relative predictive weights to the individual sensing modalities. Establishing such weights would require a trained multivariate prediction model followed by appropriate feature-importance or ablation analysis, neither of which was part of the present experimental design. The individual analyses did not identify GSR-derived skin resistance, surface temperature, or RGB skin color as a sufficiently strong standalone predictor of fingerprint image quality. This finding does not imply that these modalities are uninformative; rather, their predictive relevance may depend on interactions among skin properties, environmental conditions, sensing technology, and morphological degradation of the friction-ridge surface. Determining the relative contributions of individual modalities and their combinations therefore remains an objective for future multivariate models trained and independently validated on larger and appropriately labeled datasets.

The present post-mortem study therefore supports the practical relevance of objective skin characterization for skin-aware fingerprint acquisition while also identifying an important limitation of the current multisensor system: it characterizes the optical, electrical, and thermal state of the skin but does not directly quantify the morphology of the friction-ridge surface. Future development should therefore complement the existing measurements with direct assessment of skin-surface morphology; for example, through image-based, 3D, or depth-sensing methods capable of quantifying wrinkling, ridge folding, flattening, epidermal sloughing, and other geometrical or decomposition-related alterations. These morphological descriptors could subsequently be combined with the existing skin and environmental measurements, sensing-technology information, and fingerprint image-quality measures within a multivariate decision-support model for skin conditioning, sensor selection, and acquisition strategy.

### 4.7. Generative AI for Reconstruction of Degraded Fingerprints

The following analysis presents two selected degraded post-mortem fingerprints as illustrative forensic case studies and should not be interpreted as a general performance evaluation or validation of generative fingerprint enhancement. The cases were selected to illustrate both the potential improvement in fingerprint image-quality metrics and the forensic limitations associated with generative reconstruction. In particular, an increase in an objective image-quality score does not demonstrate that the reconstructed ridge pattern faithfully preserves the identity-bearing information contained in the original fingerprint.

Accordingly, this section is not intended as a comparative benchmark of fingerprint enhancement or reconstruction algorithms. Established filtering, ridge-reconstruction, orientation-guided, and deep-learning enhancement methods are therefore outside the scope of the present experimental comparison. The purpose of the analysis is instead to illustrate the distinction between improvement in fingerprint image quality and preservation of biometric fidelity when generative reconstruction is applied to severely degraded fingerprint evidence.

Generative artificial intelligence differs fundamentally from conventional fingerprint enhancement in its ability to synthesize plausible image content. Whereas conventional enhancement methods are generally designed to emphasize, regularize, or recover fingerprint structures supported by information present in the acquired image, a generative model may synthesize plausible ridge trajectories and local ridge structures in regions where the original friction-ridge information is weak, interrupted, distorted, or unavailable. This capability may improve the visual appearance and image-quality characteristics of severely degraded post-mortem fingerprints, but it also introduces a critical limitation for forensic applications: synthetically generated ridge structures cannot automatically be regarded as recovered biometric evidence.

For the two illustrative cases, the original fingerprint images contained recognizable friction-ridge patterns, but local ridge continuity and ridge–valley separability were substantially affected by post-mortem skin condition and deformation. Generative reconstruction was applied to these images to examine whether the resulting fingerprints exhibited improved image-quality characteristics and, at the same time, to demonstrate why such improvement must be evaluated separately from preservation of the original ridge topology and identity-bearing biometric information.

Conceptually, the generative reconstruction can be represented as

(10) $$I_{\mathrm{GAN}}=G\left(I_{\mathrm{raw}},O,F,M\right),$$

where $I_{\mathrm{raw}}$ is the original fingerprint image, G(·) denotes the generative reconstruction model, O represents the estimated local ridge-orientation field, F describes local ridge-frequency information, and M identifies regions containing weak, corrupted, or unreliable fingerprint information.

The processing aims to generate a spatially coherent ridge–valley pattern consistent with the dominant ridge flow visible in the original image. Following generative reconstruction, intensity normalization and local ridge–valley contrast adjustment can additionally be applied. Importantly, however, the generated image must be interpreted as a *synthetic reconstruction* rather than as a recovery of information that was demonstrably present in the original fingerprint.

**Figure 7:**
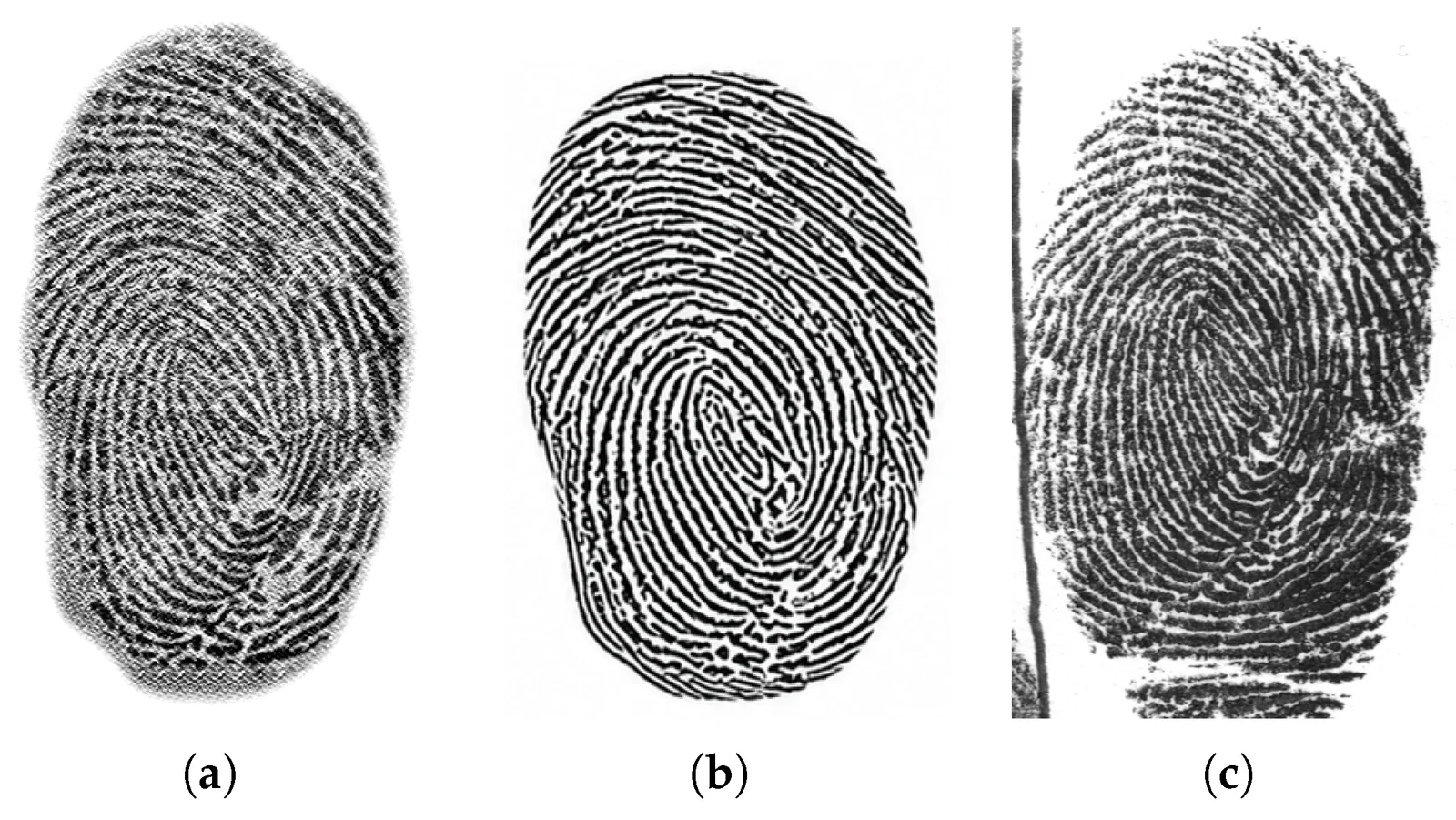
Examples of post-mortem fingerprints: (**a**) fingerprint acquired using the Jenetric LiveTouch Duo scanner; (**b**) fingerprint after generative enhancement; and (**c**) reference inked fingerprint from a fingerprint card.

The first representative example is shown in Figure 7. At the time of skin assessment, the corresponding multisensor measurements were

(11) $$T_{\mathrm{skin},1}=15\;{}^{\circ}C,\;GSR_{1}=1920\;\Omega ,$$

with the compensated skin-color measurement

(12) $$c_{\mathrm{RGB},1}={\left[0.75,\;0.77,\;0.63\right]}^{T}.$$

These values characterize the physical condition of the skin associated with the original fingerprint acquisition and are not inputs to the generative reconstruction presented here.

The corresponding NFIQ 2 quality scores were

(13) $$Q_{\mathrm{NFIQ},1}^{\left(a\right)}=23,\;Q_{\mathrm{NFIQ},1}^{\left(b\right)}=63,\;Q_{\mathrm{NFIQ},1}^{\left(c\right)}=38.$$

The generative reconstruction therefore increased the NFIQ 2 score from 23 to 63, corresponding to an absolute increase of 40 points. Remarkably, the generated image also received a substantially higher quality score than the visually high-quality reference ($Q_{\mathrm{NFIQ}}=38$). From the perspective of automated image-quality assessment, the generative processing was therefore highly successful.

**Figure 8:**
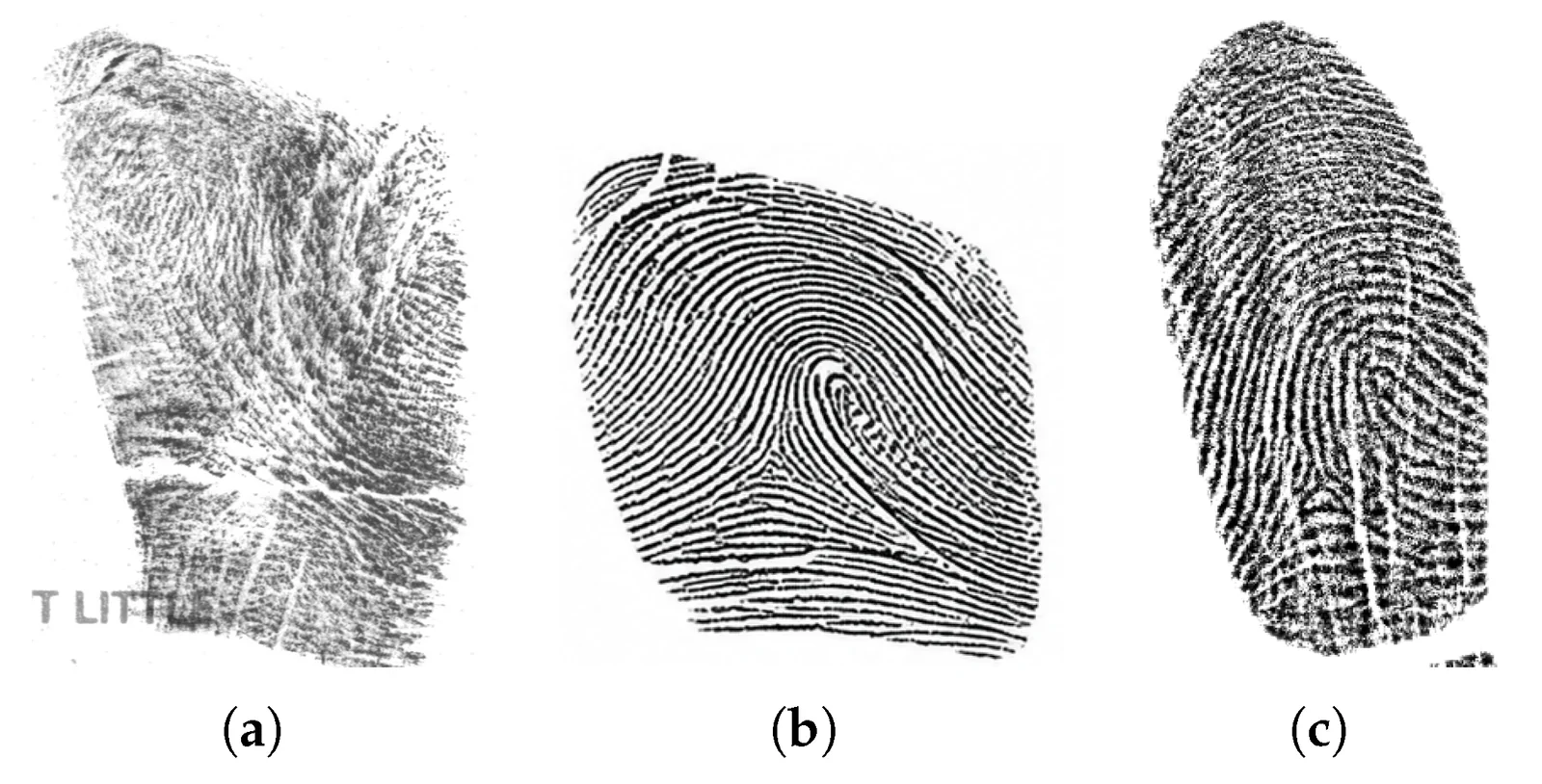
Second illustrative forensic case of generative fingerprint enhancement: (**a**) damaged post-mortem inked fingerprint from a fingerprint card; (**b**) generatively enhanced image; and (**c**) contactless IDloop acquisition of the same finger used for visual reference. The example illustrates the potential improvement in image quality as well as the risk of introducing ridge structures not unambiguously supported by the original evidence.

The second example represents a more severely degraded friction-ridge surface and is shown in Figure 8. The associated skin measurements were

(14) $$T_{\mathrm{skin},2}=8\;{}^{\circ}C,\;GSR_{2}=2450\;\Omega ,$$

and

(15) $$c_{\mathrm{RGB},2}={\left[0.73,\;0.90,\;0.55\right]}^{T}.$$

The corresponding NFIQ 2 scores were

(16) $$Q_{\mathrm{NFIQ},2}^{\left(a\right)}=10,\;Q_{\mathrm{NFIQ},2}^{\left(b\right)}=52,\;Q_{\mathrm{NFIQ},2}^{\left(c\right)}=24.$$

Here, the generative reconstruction increased the NFIQ 2 score from 10 to 52, an absolute improvement of 42 points. Again, the generated fingerprint received a considerably higher quality score than the visually high-quality reference ($Q_{\mathrm{NFIQ}}=24$). In the second illustrative case, the NFIQ 2 score increased from 10 for the damaged inked fingerprint to 52 after generative enhancement. This substantial numerical improvement indicates that the reconstructed image exhibits image characteristics that are more favorable according to the NFIQ 2 quality model; however, it must not be interpreted as evidence of improved forensic fidelity. Visual comparison indicates that the generative process may alter local ridge topology by introducing ridge connections, endings, bifurcations, or trajectories that cannot be unambiguously supported by the original fingerprint. Consequently, the enhanced image should be regarded as a synthetic reconstruction suitable for illustrating the potential of generative processing, rather than as a substitute for the original fingerprint evidence in forensic identification. A formal minutiae-level correspondence analysis between the original and generatively enhanced fingerprints was not performed in the present illustrative experiment. Such an analysis, including the identification of preserved, removed, displaced, and newly generated minutiae, represents an important next step for evaluating the forensic reliability of generative fingerprint reconstruction.

#### 4.7.1. Image Quality Versus Biometric Fidelity

The two experiments reveal an important limitation of generative fingerprint enhancement. In both cases, the generatively reconstructed images achieved the highest NFIQ 2 scores among the three compared images. The improvement was substantial: from 23 to 63 in the first example and from 10 to 52 in the second. Generative models can therefore produce ridge–valley structures that are highly favorable from the perspective of an automated fingerprint quality estimator.

However, *high fingerprint image quality is not equivalent to preservation of biometric identity*. Visual comparison of the original and generatively reconstructed fingerprints demonstrates that the generated ridge pattern does not necessarily correspond to the original friction-ridge structure. Where the input contains missing or ambiguous information, the generative model estimates a plausible continuation based on the available image information and the structural patterns represented by the model. It can consequently create new ridge connections, endings, bifurcations, or local ridge trajectories that are not demonstrably supported by the original acquisition.

This observation explains the apparently paradoxical NFIQ 2 results. The generative model can synthesize a regular, continuous, high-contrast ridge–valley pattern and thereby substantially increase the objective quality score, while simultaneously modifying identity-bearing information. In other words, the model can generate an image that is *better as a fingerprint image* according to a quality estimator, but not necessarily *more faithful to the fingerprint of the examined individual*. The substantial NFIQ 2 improvements observed here should therefore be interpreted primarily as evidence that the generated images possess characteristics favored by the quality estimator, rather than as evidence of successful recovery of the original biometric information.

The multisensor measurements provide an additional perspective on this problem. Skin temperature, RGB color, and GSR characterize the physical state of the finger during acquisition and may help explain why the original fingerprint was difficult to capture. They cannot, however, determine the true trajectory of a ridge that is absent from the acquired image. Consequently, these physiological measurements are suitable for predicting acquisition conditions, selecting a sensing technology, recommending skin conditioning, or parameterizing conservative enhancement, but they cannot serve as evidence for reconstructing missing identity-bearing ridge details.

For forensic applications, unrestricted generative reconstruction should therefore not be considered a restoration of the original biometric evidence. The original fingerprint must always remain the primary evidence, while a generatively reconstructed image should be explicitly labeled as synthetic and retained only as a derived representation. Its usefulness should be evaluated not only by NFIQ 2 but also by ridge-orientation consistency, minutiae preservation, genuine matching scores, and the stability of extracted biometric features.

The two examples presented in this section are intended solely as illustrative forensic cases and do not constitute a biometric validation of the generative reconstruction method. Although NFIQ 2 provides an objective indication of image characteristics associated with fingerprint utility, an increase in NFIQ 2 alone cannot demonstrate preservation of identity-bearing information. A rigorous forensic evaluation would require direct comparison of the original and generated fingerprints against an independent reference using biometric matcher scores, together with minutiae correspondence, ridge-orientation consistency, and feature-stability analysis. In particular, comparison of original-to-reference and generated-to-reference matching scores and the proportion of corresponding minutiae would provide a direct quantitative measure of whether an apparent improvement in image quality is accompanied by preservation of biometric identity. These analyses were not performed in the present illustrative experiment and therefore no claim regarding improved biometric recognition performance or forensic fidelity is made on the basis of the generated examples.

#### 4.7.2. Implications for the Proposed AI-Assisted Framework

The present findings motivate a hierarchical approach to the use of artificial intelligence within a future skin-aware fingerprint acquisition framework, rather than relying primarily on generative reconstruction. At the pre-acquisition stage, AI-/ML-based decision support could integrate skin, environmental, morphological, and acquisition-related information to reduce the likelihood of poor fingerprint acquisition. If an unfavorable skin condition or acquisition context is identified, the preferred response should be to preserve and acquire genuine biometric information through controlled skin conditioning, selection of a more appropriate sensing technology, or reacquisition whenever possible.

If satisfactory reacquisition is impossible or unsuccessful, conservative AI-based enhancement may subsequently be considered, preferably using methods constrained by information supported by the acquired fingerprint, including ridge orientation, segmentation, minutiae, and local ridge topology. Generative reconstruction should represent the final and most carefully controlled processing stage because of its greater potential to synthesize plausible biometric structures that are not unambiguously supported by the original fingerprint evidence.

The proposed processing hierarchy can therefore be summarized as

(17) $$\begin{array}{cc} x_{\mathrm{skin}},x_{\mathrm{environment}},x_{\mathrm{morph}} & \to \mathrm{AI}/\mathrm{ML}\;\mathrm{decision}\;\mathrm{support}\\ \; & \to \mathrm{conditioning}/\mathrm{sensor}\;\mathrm{selection}\\ \; & \to \mathrm{acquisition}\to Q_{\mathrm{NFIQ}}\\ \; & \to \mathrm{reacquisition}\to \mathrm{conservative}\;\mathrm{enhancement}\\ \; & \to \mathrm{generative}\;\mathrm{reconstruction}. \end{array}$$

The illustrative cases presented in this study demonstrate both the potential and the principal forensic limitation of generative fingerprint reconstruction. Generative processing can substantially improve numerical image-quality scores for severely degraded fingerprints; however, such improvement does not establish that the resulting ridge pattern more faithfully represents the original biometric information. On the contrary, an improved quality score may partly reflect synthetically reconstructed ridge structures whose correspondence to the original friction-ridge topology cannot be established from the degraded input image alone. For biometric and forensic applications, preservation of identity-bearing information must therefore take precedence over visual appearance or maximization of an individual fingerprint image-quality metric.

For forensic applications in which processed images are intended to support subsequent biometric comparison, future processing pipelines should preferentially employ constrained enhancement methods designed to preserve ridge orientation, minutiae, and local ridge topology rather than unconstrained generative reconstruction. Orientation-guided processing and deep-learning architectures incorporating explicit structural or topological constraints represent promising directions; however, their forensic suitability must be established through direct biometric-fidelity evaluation, including minutiae correspondence, ridge-orientation consistency, feature-stability analysis, and matcher-based comparison against independent reference fingerprints. Any generatively reconstructed fingerprint retained for forensic purposes should additionally be explicitly identified as a synthetic derived representation and clearly distinguished from the original fingerprint evidence.

## 5. Conclusions

This study introduced and experimentally evaluated a skin-aware approach to fingerprint acquisition based on objective characterization of the physical condition of the examined skin. A portable multisensor system was developed to measure complementary optical, electrical, and thermal skin properties together with relevant environmental parameters. The approach was evaluated under real forensic conditions on 50 deceased individuals using multiple fingerprint acquisition technologies. Numerical multisensor records were available for 47 subjects, providing an experimental basis for investigating the relationships among post-mortem skin condition, sensing technology, and fingerprint image quality.

The experiments demonstrated substantial variability in the condition of post-mortem friction-ridge skin. Skin resistance, surface temperature, and optical characteristics varied considerably among the examined subjects as a consequence of heterogeneous post-mortem and examination conditions, including refrigeration, drying, washing, and exposure to the autopsy-room environment. Excessively dry or moist skin frequently complicated fingerprint acquisition, whereas simple controlled conditioning followed by reacquisition could considerably improve problematic acquisitions. At the same time, none of the individually measured parameters, including RGB skin color, GSR-derived skin resistance, or surface temperature, showed a sufficiently strong relationship with fingerprint image quality to serve as a universal standalone predictor. These findings indicate that the physical condition of the skin is relevant to fingerprint acquisition but cannot be adequately characterized for this purpose by any single measured variable.

An important finding was the role of friction-ridge surface morphology. Poor fingerprint acquisition was frequently associated with wrinkling, folding, ridge flattening, and geometrical deformation of the post-mortem skin, even when the measured optical, electrical, or thermal parameters were within otherwise unremarkable ranges. The current multisensor system does not directly quantify these morphological alterations. Consequently, a comprehensive skin-aware acquisition strategy should combine optical, electrical, thermal, and environmental measurements with direct information about the morphology of the friction-ridge surface and with sensor-specific fingerprint image-quality information. Such a multidimensional representation could provide a more appropriate basis for future decisions concerning skin conditioning, sensing-technology selection, and reacquisition than any individual skin parameter alone.

The illustrative generative-reconstruction experiments further demonstrated the importance of distinguishing fingerprint image quality from biometric fidelity. In the two presented cases, generative processing increased NFIQ 2 from 23 to 63 and from 10 to 52, respectively, indicating substantial improvement according to the image-quality metric. However, an improved NFIQ 2 score does not establish that the reconstructed ridge pattern faithfully preserves the original identity-bearing information. Generative models may synthesize plausible ridge trajectories, endings, bifurcations, or connections where the acquired image does not contain sufficient information to determine the original friction-ridge structure. For forensic applications, such synthetic structures cannot be interpreted as recovered biometric evidence. The original fingerprint must therefore remain the primary evidence, and any generatively reconstructed image retained for forensic purposes should be explicitly identified as a synthetic or AI-generated derived representation and clearly distinguished from the original fingerprint.

These observations support a hierarchical approach to future skin-aware fingerprint acquisition and processing. The primary objective should be to maximize the acquisition of genuine biometric information rather than to reconstruct missing information after capture. Objective assessment of the skin and acquisition environment should therefore first support appropriate skin conditioning and selection of a suitable sensing technology, followed by reacquisition whenever the initial fingerprint is inadequate. If satisfactory reacquisition is impossible or unsuccessful, conservative enhancement methods designed to preserve ridge orientation, minutiae, and local ridge topology should be preferred. Generative reconstruction should be considered only as a final and carefully controlled processing stage, with preservation of identity-bearing information taking precedence over visual appearance or maximization of an individual image-quality metric.

The present study nevertheless has several important limitations. The experimental cohort was relatively small and consisted exclusively of post-mortem subjects examined under specific forensic and environmental conditions; the observed relationships therefore cannot be assumed to generalize directly to living subjects, larger populations, or other acquisition environments. The current multisensor platform characterizes optical, electrical, and thermal skin properties but does not directly measure surface topography, mechanical deformation, epidermal sloughing, or other morphological and decomposition-related alterations of the friction-ridge skin. PPG-derived blood-oxygenation and heart-rate measurements were not evaluated because active peripheral circulation was absent in the post-mortem cohort. In addition, no adaptive AI/ML model for skin conditioning, sensor selection, or acquisition control was trained or validated in the present study. The generative examples were intended as illustrative forensic cases rather than as a comparative benchmark or biometric validation of a fingerprint reconstruction algorithm. Consequently, the present findings should be interpreted as an experimental evaluation of objective multisensor skin characterization under the investigated post-mortem forensic conditions rather than as validation of a complete automated skin-aware decision-support system.

To place the present findings in the context of recent developments, Table 3 compares the present study with selected fingerprint studies published between 2024 and 2026. The selected studies address complementary stages of the fingerprint acquisition and processing pipeline, including contactless fingerprint quality assessment, the influence of moisture and illumination on optical acquisition, and generative reconstruction of degraded fingerprints. Because their objectives, datasets, sensing technologies, and evaluation criteria differ substantially, the reported numerical results should not be interpreted as direct performance benchmarks against the present multisensor approach. Instead, the comparison highlights the specific contribution of objective pre-acquisition skin characterization and its potential integration with subsequent quality assessment and fingerprint processing.

Based on the present findings, identified limitations, and comparison with recent research, the principal directions for future development can be summarized as follows:

- **Broader experimental validation.** The multisensor dataset should be expanded to larger and more heterogeneous cohorts, including living participants and a broader range of environmental and occupational conditions. Such studies are necessary to assess the generalizability of the observed relationships and would additionally enable systematic evaluation of PPG-derived parameters such as heart rate and blood oxygenation. Future experimental protocols should also incorporate stricter automated data-logging and completeness-control procedures to minimize missing multisensor records.
- **Direct characterization of friction-ridge morphology.** The sensing platform should be extended with dedicated characterization of the friction-ridge surface; for example, through 3D or depth-based imaging or image-analysis methods capable of quantifying wrinkling, ridge folding, flattening, epidermal sloughing, and other geometrical or decomposition-related alterations. These morphological descriptors could complement the existing optical, electrical, thermal, and environmental measurements.
- **Multivariate AI/ML decision support.** Larger datasets with explicitly defined ground-truth acquisition outcomes should be used to train and independently validate models that combine skin, environmental, morphological, sensor-specific, and fingerprint image-quality information. Interpretable approaches such as random forests or gradient-boosted decision trees provide suitable candidates for initial investigation, while larger and more heterogeneous datasets may eventually support neural-network or multimodal information-fusion approaches, conceptually consistent with recent developments in heterogeneous multimodal data integration [37]. Such models could support skin-conditioning recommendations, sensing-technology selection, and acquisition strategy, but their effectiveness must be established through dedicated training and independent validation.
- **Biometric fidelity-preserving fingerprint enhancement.** Future enhancement studies should prioritize methods explicitly designed to preserve ridge orientation, minutiae, and local ridge topology. Orientation-guided or structurally constrained processing should be evaluated using direct biometric-fidelity measures, including minutiae correspondence, ridge-orientation consistency, feature stability, and biometric matcher scores against independent reference fingerprints. Generative reconstruction should not be considered for forensic use solely on the basis of improved visual appearance or fingerprint image-quality scores.

Training a supervised model for sensor selection or skin-conditioning recommendation will additionally require explicitly defined ground-truth labels representing the optimal acquisition strategy for each case; such labels were not established in the present experimental protocol. The development of such ground truth is therefore an essential methodological requirement for progressing from the present multisensor characterization toward a validated predictive decision-support system.

Overall, the present study demonstrates the feasibility and practical relevance of objective multisensor characterization of skin condition for fingerprint acquisition and shows that skin condition, sensing technology, friction-ridge morphology, and fingerprint image quality should be considered as interacting components rather than as independent factors. The results provide an experimental foundation for a future skin-aware acquisition framework in which objective pre-acquisition assessment supports conditioning, sensor selection, and reacquisition before increasingly interventionist image-processing methods are considered. The integration of these measurements into a validated AI-/ML-based decision-support system remains a direction for future research, with acquisition and preservation of genuine identity-bearing information as the principal requirement for biometric and forensic use.

## Figures and Tables

**Table 1:** Summary of selected environmental and skin parameters for cases with available numerical multisensor records (n=47).

Parameter	Mean	SD	Median	Min.	Max.
Ambient temperature (°C)	23.54	1.46	23.40	20.40	26.50
Relative humidity (%)	47.74	9.16	48.00	29.00	64.00
Finger skin resistance (kΩ)	998.33	844.20	705.00	46.00	3294.00
Finger temperature (°C)	10.52	8.22	11.50	−11.70	30.30
Inguinal temperature (°C)	9.40	5.80	8.50	−2.00	27.10

**Table 2:** Summary of compensated RGB skin-color measurements at the finger and inguinal reference site (n=47). Differences are defined as Δ=finger−inguinal and RGB values are normalized to the interval [0,1].

Parameter	Minimum	Median	Maximum
Finger *R*	0.670	0.810	0.884
Finger *G*	0.600	0.790	0.920
Finger *B*	0.580	0.730	0.920
Inguinal *R*	0.620	0.827	0.930
Inguinal *G*	0.570	0.820	0.950
Inguinal *B*	0.460	0.730	0.927
ΔR	−0.178	−0.020	0.190
ΔG	−0.240	−0.030	0.240
ΔB	−0.185	0.010	0.230

**Table 3:** Contextual comparison with selected recent fingerprint studies published in 2024–2026. The studies address complementary aspects of fingerprint acquisition, quality assessment, and enhancement and therefore do not constitute direct performance benchmarks.

Study	Focus	Main Findings	Relation to the Present Study
Priesnitz et al. (2024) [34]	Contactless fingerprint quality assessment	MCLFIQ improved quality prediction for mobile contactless fingerprints compared with NFIQ 2 and a sharpness-based measure.	Addresses post-acquisition quality assessment; the present study additionally characterizes skin condition before acquisition.
Shin et al. (2025) [35]	Effects of moisture and illumination on FTIR acquisition	Fingerprint quality was influenced by fingertip moisture and illumination wavelength; wet conditions and green/blue illumination showed favorable quality characteristics in the investigated cohort.	Supports the relevance of skin condition and sensor-specific acquisition conditions investigated by the present multisensor approach.
Xie et al. (2026) [36]	Generative reconstruction of degraded fingerprints	Ridge-flow-guided reconstruction increased median NFIQ 2 from 9 to 44, minutiae-restoration F1-score from 0.2507 to 0.5426, and Rank-1 identification from 39% to 86%.	Demonstrates the importance of evaluating generative reconstruction using biometric-fidelity and recognition measures in addition to image quality.
Present study	Pre-acquisition multisensor skin characterization	No individual measured skin parameter was a universal predictor of fingerprint quality; controlled skin conditioning could improve problematic acquisition.	Integrates objective skin assessment with sensor-dependent acquisition and explicitly distinguishes fingerprint image quality from biometric fidelity.

## Data Availability

The data generated and analyzed during this study are available from the corresponding author upon reasonable request. Access may be granted for justified non-commercial research purposes, subject to agreement on the intended use of the data and compliance with applicable ethical, legal, and data-protection requirements.

## Abbreviations

The following abbreviations are used in this manuscript:

ADC	Analog-to-Digital Converter
AI	Artificial Intelligence
BPM	Beats Per Minute
CCM	Color Correction Matrix
CNN	Convolutional Neural Network
CSV	Comma-Separated Values
FTIR	Frustrated Total Internal Reflection
GAN	Generative Adversarial Network
GDPR	General Data Protection Regulation
GSR	Galvanic Skin Response
MEMS	Micro-Electro-Mechanical Systems
ML	Machine Learning
NFIQ	NIST Fingerprint Image Quality
NIST	National Institute of Standards and Technology
NIR	Near-Infrared
PPG	Photoplethysmography
RGB	Red Green Blue
RTC	Real-Time Clock
SD	Standard Deviation
USB	Universal Serial Bus
